# Application of machine learning techniques to tuberculosis drug resistance analysis

**DOI:** 10.1093/bioinformatics/bty949

**Published:** 2018-11-21

**Authors:** Samaneh Kouchaki, Yang Yang, Timothy M Walker, A Sarah Walker, Daniel J Wilson, Timothy E A Peto, Derrick W Crook, CRyPTIC Consortium, David A Clifton

**Affiliations:** 1 Department of Engineering Science, Institute of Biomedical Engineering; 2 Nuffield Department of Medicine, University of Oxford; 3 National Institute of Health Research Oxford Biomedical Research Centre, John Radcliffe Hospital, Oxford, UK; 4 Medical Research Council Clinical Trials Unit, University College London, UK; 5 Nuffield Department of Population Health, Big Data Institute, University of Oxford, Li Ka Shing Centre for Health Information and Discovery, Oxford, UK; 6 National Infection Service, Public Health England, Colindale, London, UK; 7 A Corporate Author; for the list of members, please see the section at the end of this article

## Abstract

**Motivation:**

Timely identification of *Mycobacterium tuberculosis* (MTB) resistance to existing drugs is vital to decrease mortality and prevent the amplification of existing antibiotic resistance. Machine learning methods have been widely applied for timely predicting resistance of MTB given a specific drug and identifying resistance markers. However, they have been not validated on a large cohort of MTB samples from multi-centers across the world in terms of resistance prediction and resistance marker identification. Several machine learning classifiers and linear dimension reduction techniques were developed and compared for a cohort of 13 402 isolates collected from 16 countries across 6 continents and tested 11 drugs.

**Results:**

Compared to conventional molecular diagnostic test, area under curve of the best machine learning classifier increased for all drugs especially by 23.11%, 15.22% and 10.14% for pyrazinamide, ciprofloxacin and ofloxacin, respectively (*P* < 0.01). Logistic regression and gradient tree boosting found to perform better than other techniques. Moreover, logistic regression/gradient tree boosting with a sparse principal component analysis/non-negative matrix factorization step compared with the classifier alone enhanced the best performance in terms of F1-score by 12.54%, 4.61%, 7.45% and 9.58% for amikacin, moxifloxacin, ofloxacin and capreomycin, respectively, as well increasing area under curve for amikacin and capreomycin. Results provided a comprehensive comparison of various techniques and confirmed the application of machine learning for better prediction of the large diverse tuberculosis data. Furthermore, mutation ranking showed the possibility of finding new resistance/susceptible markers.

**Availability and implementation:**

The source code can be found at http://www.robots.ox.ac.uk/ davidc/code.php

**Supplementary information:**

Supplementary data are available at *Bioinformatics* online.

## 1 Introduction

Tuberculosis (TB) is one of the leading causes of mortality across the world (World Health Organization *et al.*, 2016). In 2016, there were 600 000 new cases with resistance to rifampicin (RIF), the most effective first-line drug, including 490 000 cases of multi-drug-resistant TB (MDR-TB) (World Health Organization *et al.*, 2016). Hence, TB drug resistance is an urgent public health concern in the field of infectious disease. In TB, drugs are usually grouped into first-line drugs [isoniazid (INH), RIF, ethambutol (EMB) and pyrazinamide (PZA)] and second line drugs [streptomycin (SM), fluoroquinolones-ofloxacin (OFX), moxifloxacin (MOX), ciprofloxacin (CIP), kanamycin (KAN), amikacin (AK) and capreomycin (CAP)] (Jnawali and Ryoo, 2013). Conventional whole genome sequencing (WGS) methods are based on identifying a number of variants (i.e. single nucleotide polymorphisms, insertions or deletions) and interpreting them as associated (or not) with conferring resistance to each individual drug (Schleusener *et al.*, 2017). Hence, they relies on a library of previously identified resistance-associated variants (Coll *et al.*, 2015; Georghiou *et al.*, 2012; Walker *et al.*, 2015). Such techniques can result in lower performance particularly for less-studied drugs e.g. PZA and second-line drugs due to high dimensionality and improper composition of the library.

In addition to methods based on known mutations, machine learning models have been applied to determine drug resistance, e.g. logistic regression (LR), support vector machine (SVM) and random forest (RF) (Farhat *et al.*, 2016; Yang *et al.*, 2018; Zhang *et al.*, 2013). Such models have been shown to perform similarly to the variant-based association rules for well-studied drugs, e.g. INH, RIF and EMB while outperforming them for less-studied drugs, e.g. PZA. However, to date few studies have investigated machine learning methods for TB resistance prediction and they have used a limited number of isolates. Zhang *et al.* (Zhang *et al.*, 2013) used LR to investigate 161 isolates from China to try to discover new genes associated with resistance to seven drugs. Yang *et al.* (Yang *et al.*, 2018) considered 1839 UK bacterial isolates and compared a number of classification models for eight drugs (CAP, AK and KAN excluded from their analysis due to insufficient resistant samples). Farhat *et al.* (Farhat *et al.*, 2016) used a more geographically diverse dataset to investigate the performance of RF using 1397 isolates. Considering a small dataset from a limited community can lead to over-fitted models. Although considering cross-validation and regularization terms can help with over-fitting, a larger more diverse dataset should be considered to confirm the performance of them for resistance prediction and also have a more general trained model that can better predict the future samples. In addition, as the feature space dimensionality grows, it becomes sparser and sparser (as the high dimensional genomic information from WGS). Consequently, fitting a separable hyperplane can be easier but the classifier then tries to learn the specific instances and outliers of the training dataset. It then could fail to perform well for the unseen data. Furthermore, dimension reduction techniques can be used to reduce the curse of dimensionality, noise and improve the computational cost.

Here, our aim is to confirm the application of machine learning methods considering a more general dataset and also to check the effect of reducing the dimension on final results. Hence, as an extension of previous work, a number of machine learning models were developed and evaluated for resistance prediction. We studied a database of 13 402 isolates that is a more diverse and much larger dataset compared to reported machine learning TB studies. Similar to previous work, this dataset has some missing data and more susceptible isolates than resistant ones (in particular, being highly imbalanced for some drugs i.e. CAP and AK). Moreover, some other ensemble learning techniques were developed and compared with the basic machine learning and RF models mainly used in other studies (Farhat *et al.*, 2016; Yang *et al.*, 2018; Zhang *et al.*, 2013). These methods were considered here as they have been shown to be accurate and effective classification models in several applications in other areas (Ehrentraut *et al.*, 2016; Li *et al.*, 2018). Moreover, they reduce the variance by considering several independent/sequentially-built learners that are especially useful for the complex dataset in this study. Subsequently, highly ranked features from the top performing classifiers were represented and compared with the library of known mutations. The effect of two linear dimension reduction techniques on performance was also investigated especially for less-studied drugs in which not all resistance-associated mutations are known. As a summary, our results confirm the application of machine learning algorithms to drug resistance prediction for the diverse TB dataset considered here. Moreover, results show that ranked variants include known resistance/susceptible markers, resistance co-occurrence, lineage associated mutations and unknown mutations possibly as new resistance/susceptible markers.

## 2 Materials and methods

### 2.1 WGS and drug susceptibility test

For details of DNA sequencing refer to the work presented by (CRyPTIC Consortium and the 100 000 Genomes Project, 2018) and (Walker *et al.*, 2015). Sequenced reads were aligned to the reference *Mycobacterium tuberculosis* (MTB) strain. Then nucleotide bases were filtered based on the sequencing and alignment quality and per base coverage. Hence, low confident nucleotide bases were denoted as null calls and not considered in our analysis. We had several ways to treat a null call in an isolate: (i) remove the sample completely from the analysis which drastically reduce the sample size (34% of isolates have one or more null calls in the genetic regions of interest) and generalizability, (ii) consider the null calls as no variant (i.e. 0) which is a conservative option and means that performance will be an underestimate of true performance if all variants known and (iii) consider null values as missing and impute their values, either singly or multiply. We chose the second option (assume absence of variant) because the total number of positions across the genetic regions of interest (5919 positions) across all isolates (13 402) with null calls were very small [150958/79326438(=5919 × 13402) (0.19%)] and because of the complexity of multiple imputation models based on the 5919 positions. (ii) Is effectively a single hard (conservative) imputation. On all isolates, drug susceptibility testing was performed for up to 11 drugs through an initial phenotypic drug susceptibility testing using culture and confirmed using Lowenstein Jensen methods.

### 2.2 Baseline methods

Existing baseline methods classify drug resistance as present or absent based on a number of predetermined library of variants from the literature. The method denoted direct association (DA) uses an ‘OR’ rule to classify an isolate against a given drug: the isolate is labelled as resistant if any of its mutations is a resistant variants. Otherwise, it is classified as susceptible if only susceptible variants exist in the isolate. The library described by Walker *et al.* in 2015 (Walker *et al.*, 2015) was used throughout the classification comparison.

### 2.3 Linear dimension reduction

Dimensionality reduction plays an important role in machine learning mainly for a dataset with thousands of features, such as the TB data. Moreover, they have been shown to improve classification performance in many applications by reducing unimportant and redundant features (Benetos *et al.*, 2006; Malhi and Gao, 2004). Principal components analysis (PCA) as a common linear dimensionality reduction technique, is easy to understand and use in real applications and also helps to improve the classification results. It projects the data into a lower dimensional space using singular value decomposition (SVD), $X=U\Sigma V^{T}$ where **U** and **V** are singular vectors and Σ represents singular values (Eckart and Young, 1936; Golub and Van Loan, 2012). Input to SVD can have mixed-signs and there is no constraint on the factors’ signs. Non-negative matrix factorization (NMF) restricts factors to be non-negative and can be used when the input data is non-negative. Hence, it works based on putting a non-negativity constraint on the extracted components, $X_{+}=W_{+}H_{+}$ where ${\left(.\right)}_{+}$ represents the non-negativity of all elements of the input data and also components. Binary matrix factorization is another extension of NMF for the binary data by constraining components to be binary, $X_{0-1}=W_{0-1}H_{0-1}$ where ${\left(.\right)}_{0-1}$ represents the binary elements. Sparsity constraints (by constraining factorized components’ norm) can be added to the optimization of PCA and NMF to enhance interoperability and stability of components (SPCA/SNMF) (Hoyer, 2004; Zou *et al.*, 2006). Sparsity constraints are particularly important for our data due its sparse nature. Here, our results focus on linear techniques; as adding the binary constraint did not improve our results Supplementary Material I, only SPCA and SNMF were reported. A total of 100 components were kept as experiments show that 100 components keep the maximum variance for all drugs.

### 2.4 Classification methods

We investigated three basic machine learning classifiers; SVM, LR and product-of-marginals (PM), based on the original feature space or the feature space after dimension reduction. Three ensemble learning methods, RF, Adaboost and gradient tree boosting (GBT) were also considered. Details of each method, parameter settings and pros and cons of each method are shown in Supplementary Material A.

## 3 Results

### 3.1 Data description

The dataset used in this paper contains 13 402 isolates collected from across the world. Twenty-three genes which contain previously-found resistance-associated variants (Walker *et al.*, 2015) were targeted. For each isolate, the presence/absence of a mutation was represented by a binary variable, with 1 indicating the presence and 0 indicating the absence. The mean of variants per isolate was 14, ranging between 1 and 132. In total, 5919 variants were found in the 23 candidate genes across the isolates. Hence, a binary vector of 5919 was formed and considered fully or partially for the feature space (3.2 Feature spaces). For each drug and isolate, a binary label of resistance/susceptible was considered. The phenotypic information was available for up to 11 anti-TB drugs as shown in Table 1 (not all samples were tested against all drugs, leading to missing labels).

**Table 1.** The phenotype profile; the number of isolates that are resistant or susceptible

Drug	INH	EMB	RIF	PZA	SM	KAN	AK	CAP	CIP	OFX	MOX
Susceptible	9620	11 322	10 359	9806	5105	1925	2690	2741	529	2618	1249
Resistant	3457	1571	2808	1262	1729	242	273	315	77	458	262
Total tested	13 077	12 893	13 167	11 068	6834	2167	2963	3056	606	3076	1511
Missing	325	509	235	2334	6568	11 235	10 439	10 346	12 796	10 326	11 891

Around 98% of isolates were tested for phenotypic resistance to INH and RIF, 96% for EMB, 81% for PZA, 52% for SM, less than a quarter for OFX, CAP, AK, KAN and MOX and only 4% for CIP showing more missing labels for second-line drugs. All 11 drugs had substantially more susceptible than resistant isolates; more than 87% of isolates were susceptible for EMB and PZA and 72% for INH and RIF, leading to a highly imbalanced dataset. Moreover, resistance to some drugs commonly co-occurred with others, as expected, e.g. 715 isolates were co-resistant to INH, PZA, RIF and EMB.

### 3.2 Feature spaces

Following the work presented by (Yang *et al.*, 2018) and to evaluate the performance of the different classifiers, three feature sets were considered: (i) F1 was the baseline feature space is all variants found within the 23 candidate genes, (ii) F2 was the predetermined resistance-associated variants as listed in (Walker *et al.*, 2015) (Supplementary Material G) and (iii) F3 was a subset of F1 including only resistance-associated genes for the particular drug [genes with resistance-determinants specific to each drug can be found at (Walker *et al.*, 2015)].

### 3.3 Training and testing

For all experiments, the classification was performed by training a balanced training dataset and then tested over an imbalanced dataset. This was run over 100 iterations of 5-fold cross validation. In each fold, 20% of the data was considered as the test set. Within the remaining 80% of the data, susceptible samples were sub-sampled randomly to make the number of resistant and susceptible samples equal (i.e. create a balanced set) and then split 80:20 into training and validation sets. The performance in terms of accuracy, sensitivity, specificity, F1-score and area under curve (AUC) was calculated for the validation sets (for parameter setting) and test sets (for final comparison) and averaged over iterations; mean and SD were reported.

(1) Accuracy=TP+TNTP+TN+FP+FNSensitivity=TPTP+FN, Specificity=TNTN+FPprecision=TPTP+FP, F1-score=2precision*sensitivityprecision+sensitivity,

where TP, TN, FP and FN are true positive, true negative, false positive and false negative, respectively. Considering a probability estimate as the output of each classifier for the validation set and setting various thresholds to categorize this output as resistant/susceptible could result in different TP, FP, FN and TN rates. Alternatively, a receiver operating characteristic (ROC) curve showing the sensitivity as a function of 1–specificity for different thresholds; each point in the curve indicates a specific value for sensitivity, specificity and accuracy. AUC is the area under the ROC curve. Here, the ‘internal’ cross-validation on the 80% training dataset was used to select a decision threshold that maximizes the accuracy. The parameters of the models (kernel parameters for SVM or number of ensembles for RF) were also optimized through the internal cross-validation on the train data. This was done by a grid search over a range of values and selecting parameters that generated the best AUC. The workflow of examined classifiers can be seen in Supplementary Material F.

### 3.4 Classification results

**Fig. 1.**
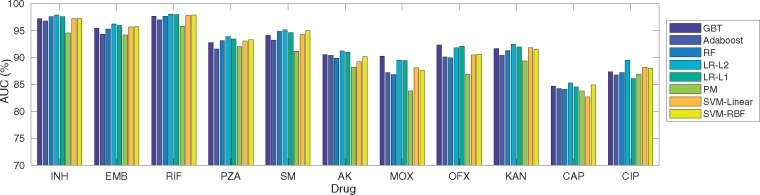
Classification performance (AUC%) considering six machine learning classifiers (LR with L1 and L2 regularization terms, SVM with Linear and RBF kernels, RF, Adaboost, PM and GBT) across 11 anti-TB drugs and F1 feature space

Figure 1 compares machine learning techniques in terms of AUC considering F1 for 11 drugs (Supplementary Material C for F1-F3). Different classifiers led to similar AUC performances except for (F1 + PM) for INH, SM, AK, MOX, OFX and KAN. AUC was much higher for PZA, MOX, OFX considering F1 compared to F2-F3, e.g. 93.89% considering F1 for PZA compared to 69.59% and 88.69% considering F2-F3 for the same drug.

**Fig. 2.**
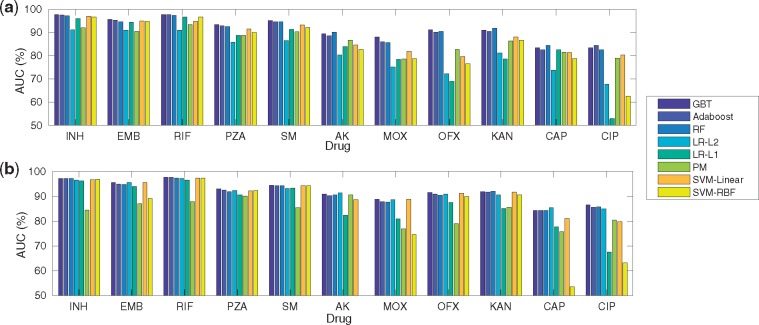
Classification performance (AUC%) considering six machine learning classifiers across 11 anti-TB drugs with (**a**) SNMF-F1 and (**b**) SPCA-F1

Figure 2 provides AUC considering (F1 + SPCA/SNMF); 100 components were considered here for all drugs. Considering SNMF led to the best performing model for INH, EMB, RIF, PZA, SM (with GBT for classification) while SPCA for AK, CAP (with LR-L2 for classification), MOX, OFX, CIP (with GBT for classification) and KAN (with RF for classification). (F1 + SNMF + PM) was performed better for RIF, SM and CAP and (F1 + SPCA + PM) for PZA and AK in comparison with (F1 + SNMF + LR-L2). With SNMF, only ensemble methods had the highest AUC for AK, MOX, OFX and KAN while with SPCA only (F1 + SPCA + PM/LR-L1) did not perform well for them. Overall, (F1 + SPCA/SNMF + GBT) was the top performing model for most drugs in terms of AUC (Fig. 2).

Table 2 provides a comparison of DA and the best performing model obtained by applying machine learning techniques on test sets considering F1–F3 for 11 drugs. Compared to DA, considering F1 with or without a dimension reduction stage improved AUC and sensitivity for all drugs, significantly (Wilcoxon signed-rank *P* < 0.01). The AUC especially increased by 23.11% for PZA, 15.22% for CIP, over 8.68% for AK, MOX, OFX and CAP and 5.16% for EMB, SM and KAN. Sensitivity increased by 44.29% for PZA, 30.42% for CIP, more than 12% for AK, MOX and OFX, 8% for EMB and KAN and 4% for SM and CAP.

**Table 2.** Comparing the best machine learning classifier and DA considering 11 drugs

Drugs	**DA**			**Best method**			
	Sensitivity	Specificity	AUC	Feature set + Classifier	Sensitivity	Specificity	AUC
INH	91.95 ± 1.04	98.71 ± 0.22	94.95 ± 0.54	F1 + LR – L2	92.19^◊^_ _± 0.94	98.38 ± 0.29	97.89^◊^_ _± 0.38
EMB	83.31 ± 1.62	95.17 ± 0.38	89.24 ± 0.85	F1 + LR – L2	92.12^◊^_ _± 0.98	91.89 ± 0.84	96.25^◊^_ _± 0.54
RIF	91.70 ± 1.19	98.73 ± 0.22	95.22 ± 0.59	F1 + LR – L2	92.27^◊^_ _± 1.25	97.45 ± 0.63	98.08^◊^_ _± 0.32
PZA	43.11 ± 2.97	98.46 ± 0.27	70.78 ± 1.46	F1 + LR – L2	88.12^◊^_ _± 2.65	88.91 ± 1.66	93.89^◊^_ _± 0.80
SM	82.80 ± 1.90	97.19 ± 0.44	89.99 ± 0.99	F1 + LR – L2	87.40^◊^_ _± 1.98	94.15 ± 1.23	95.15^◊^_ _± 0.56
AK	65.21 ± 5.32	99.70 ± 0.24	82.46 ± 2.70	F1 + SPCA + LR – L2	77.23^◊^_ _± 6.96	89.84 ± 3.05	91.37^◊^_ _± 2.36
MOX	62.97 ± 6.60	98.80 ± 0.68	80.89 ± 3.32	F1 + GBT	76.84^◊^_ _± 9.29	87.19 ± 8.21	90.27^◊^_ _± 2.96
OFX	65.07 ± 3.92	99.31 ± 0.28	82.19 ± 1.98	F1 + GBT	79.06^◊^_ _± 6.94	90.88 ± 6.38	92.33^◊^_ _± 1.49
KAN	72.31 ± 5.40	97.61 ± 0.65	84.96 ± 2.68	F1 + LR – L2	80.41^◊^_ _± 6.48	93.48 ± 4.93	92.49^◊^_ _± 2.93
CAP	59.68 ± 5.84	93.87 ± 0.88	76.78 ± 2.96	F1 + SPCA + LR – L2	64.44^◊^_ _± 6.02	92.74 ± 2.52	85.46^◊^_ _± 2.02
CIP	46.65 ± 10.10	99.24 ± 0.89	72.95 ± 5.17	F1 + LR – L2	79.86^◊^_ _± 9.98	85.37 ± 7.65	89.53^◊^_ _± 4.06

*Note*: Sensitivity, specificity and AUC (mean ± standard error) is reported. Wilcoxon signed-rank test was used to calculate the *P*-value of each method compared with the DA and ^◊^ indicate s *P* < 0.01.

Details of comparison of all methods, drugs and features can be found in Supplementary Material B. In general, using F1 improved sensitivity and AUC while specificity was lower than DA. Classification on the reduced dimension (SNMF/SPCA + F1) compared to F1 resulted in better AUC for AK and CAP and similar AUC for other drugs (Supplementary Material B). Similarly, the sensitivity increased slightly for EMB, PZA, MOX, INH and SM using dimension reduction.

F1-Score is also reported and compared with DA. The best model based on integrating dimension reduction with the classification stage improved the F1-Score more than 9% for PZA, CAP and CIP and 7% for KAN compared to considering whole F1. More details can be found in Supplementary Material C.

### 3.5 Mutation ranking

Table 3 shows the top 10 important features selected for each drug by considering the top performing models (Supplementary Material D shows the mutation for LR-L1, LR-L2 and GBT models). Mutations that were associated with resistance to the specific drug in the library are indicated in boldface. Results indicated that machine learning methods could also rank several known mutations as important especially for well-studied drugs. There are also some lineage defining mutations ranked as important for AK, OFX, KAN, CAP and CIP (indicated by ${}^{*}$ in Table 3). However, there were several mutations selected as important that were not in the library and not lineage defining some with gene related to each considered drug (for EMB, PZA, SM, AK, OFX, MOX and CAP indicated by *^o^* in Table 3). Moreover, there was high overlap between selected mutations based on all three methods for RIF, OFX and MOX, LR-L1 and LR-L2 for EMB and KAN and LR-L2 and GBT for INH and EMB. Furthermore, some mutations found to be important for more than one drug.

**Table 3.** Top 10 mutations ranked by top performing classifier for each drug

INH	EMB	RIF	PZA	SM	AK	OFX	MOX	**KAN**	**CAP**	**CIP**
**katG_S315T**	embB_Y319S*^o^*	**rpoB_S450L**	katG_S315T	katG_S315T	**rrs_A1401G**	**gyrA_D94G**	**gyrA_D94G**	**rrs_A1401G**	**rrs_A1401G**	**gyrA_D94G**
**fabG1_C-15T**	**embB_M306V**	pncA_H51D	**pncA_H57D**	**rpsL_K43R**	gyrA_E21Q^*^	**gyrA_A90V**	**gyrA_A90V**	**eis_G-10A**	**rrs_C1402T**	gyrA_D94A
**fabG1_L203L**	**embB_D328G**	**rpoB_H445Y**	**pncA_L120P**	**rpsL_K88R**	gidB_S100F^*^	**gyrA_S91P**	**gyrA_D94Y**	**eis_C-14T**	embB_R507R	pncA_D136G
**katG_S315N**	rrs_C513T	**rpoB_H445D**	katG_S315N	**rrs_A514C**	katG_S315T	**gyrA_D94A**	**gyrA_D94A**	**eis_G-37T**	gidB_G62G*^o^*	**gyrA_A90V**
rpoB_S450L	**embB_Q497R**	**rpoB_D435V**	rpsA_A381V*^o^*	**rrs_C517T**	eis_C-14T	**gyrA_G88C**	**gyrA_S91P**	gyrB_G77S	pncA_D12N	gyrA_S95T^*^
rpoB_V170F	**embA_C-16G**	**rpoB_V170F**	pncA_H51D*^o^*	inhA_I194T	pncA_T142A	**gyrA_D94N**	**gyrA_D94N**	gyrA_E21Q^*^	gyrA_E21Q^*^	embB_M306I
**fabG1_G-17T**	**embB_G406A**	**rpoB_S450W**	**pncA_G97D**	**gidB_P75R**	rpoB_C-61T^*^	**gyrA_D94H**	**gyrA_D94H**	embA_Q38Q	ndh_Y108C	**gyrA_S91P**
rpoB_D435V	**embA_C-11A**	**rpoB_H445L**	**rpsA_A440T** ^+^	rrs_G878A*^o^*	embB_Y334H	**gyrA_D94Y**	**gyrA_G88C**	embB_Y334H	iniC_T89I	katG_C-85T
rpoB_S450W	**embB_D328Y**	**rpoB_H445C**	**pncA_Q10P**	gyrA_K542K	pncA_P62S	gyrA_E21Q^*^	gyrB_D500N*^o^*	**eis_C-12T**	rpoB_C-61T^*^	rpoB_V168A
rpsA_A381V	**embB_D1024N** ^+^	**rpoB_S450F**	katG_V473L	rrs_A906G	gidB_G62G*^o^*	gyrB_E540D*^o^*	katG_S315T	pncA_H57R	pncA_F13L	embB_D869E

*Note*: Resistance/susceptible-associated mutations to each given drug are indicated in the boldface (susceptible-associated indicated by ^+^). The other mutations are either known to be related to other drugs, are lineage related (indicated by ^*^), or not in the library (indicated by *^o^*). *fabG1*_L203L is a misnomer. *fabG1* and *inhA* are basically contiguous and the L203L mutation actually acts as a mutation in the promoter region for *inhA* and increases expression of the *inhA* gene (Kandler *et al.*, 2018).

## 4 Discussion

Developing machine learning techniques is especially important to improve TB resistance prediction, mainly sensitivity for less-studied drugs. Table 2 and Supplementary Material B indicate that machine learning techniques generally improved AUC and sensitivity but resulted in lower specificity compared to a clinical algorithm (DA). This is because machine learning techniques can tune the optimal operational point to balance sensitivity and specificity, while DA cannot. The performance is especially improved for less-studied drugs, e.g. for PZA, the 2015 DA method resulted in relatively low sensitivity (43.14%), AUC (70.78%) and F1-Score (55.55%) which was improved by using (F1 + LR-L2) to 88.12%, 93.89% and 64.36%, respectively. The results indicate the effectiveness of machine learning methods is based on the input feature space. There may be several reasons for this including the existence of additional resistance-associated mutations to the ones reported in the literature or co-occurrence of resistance to multiple drugs within the 23 genes considered in this paper.

However, mutations within known genes contributed more to identify resistance to each drug. Hence, F3 feature set contains enough information to solve the classification performance in terms of F1-Score (Supplementary Material B and C). On other hand, F1 feature space resulted in higher AUC and sensitivity but lower specificity (Supplementary Material B). It could be that there are some resistance-related variants and interactions from not only genes suspected to be related to resistance of each drug, but also the remaining of the 23 genes. Lower specificity means more susceptible isolates were falsely classified to be resistant possibly due to the resistance co-occurrence pattern dominating the classification. The lower performance for CAP, MOX, AK and CIP (although still highly improved in comparison with DA) is mainly because there were very few labelled isolates especially resistant cases for them. It could be also due to the underlying resistance co-occurrence pattern.

In comparison with the former work published by (Yang *et al.*, 2018) that showed higher AUC using F1–F3 depending on the drug, our results showed that F1 had better AUC. It can mainly be due to more cases of resistance co-occurrence, existence of additional mutations to reported resistance-determinants or complex interaction of mutations for our more complex dataset. It also shows the potential of using machine learning to work directly with high dimensional WGS. Moreover, reported AUC in their work was higher for eight reported drugs except for SM compared to our reported results (Supplementary Material K) potentially as a result of considering a larger more complex dataset here and lack of generalizability of the resistance co-occurrence predictions. Moreover, LR-L1 was not among the top performing classifiers and GBT was not considered in their analysis, whereas they were top performing methods especially considering F1 for our data. One possible reason could be the diversity of the data that was available for the training stage in our data. As a result, it could lead to less over-fitting and a more generalizable conclusion. Furthermore, LR can work well with binary target, usually has low variance and is less prone to over-fitting by using regularization terms. GBT is an accurate and effective classification model in several applications and is robust to outliers in the output space by considering a robust loss function. Consequently, both methods could work for this complex and noisy dataset. In addition, only RF was considered by (Farhat *et al.*, 2016) for 13 drugs. Considering drugs analyzed in both papers, we had better sensitivity for EMB, PZA, SM, KAN, CAP and CIP and higher or similar specificity for INH, EMB and OFX (Supplementary Material K).

Regarding mutations ranking, machine learning methods ranked not only the known mutations as important but also some other mutations in genes associated with resistance to other drugs. It can be because that there are some commonly extracted mutations for some drugs due to several cases of MDR-TB and hence a limitation of the feature ranking based on the single-label drug resistance classification. It also can reflect known accumulation of drug resistance mutations as patients take second-line drugs after first-line drugs. Moreover, there are some mutations for some drugs that are not in the library as associated to that drug, are not in the list of lineage defining mutations and have genes known to be associated to that drug. Hence, they can be considered as drug-associated mutations found by machine learning methods. Consequently, it shows the application of machine learning to find new important mutations. We note if there are some highly correlated features, any of them may be selected with no preference of one over others. After selecting one of such a set of highly correlated features, the importance of other correlated features is decreased. From the point of view of classification, this is actually useful as it removes the features described by others. However, for feature selection it may lead to a conclusion that one feature is important in comparison to others while in fact they are all highly correlated. Moreover, the ranked lineage associated mutations e.g. *rpob*_C-61T appeared in lineages with a large number of isolates such as Central Asian sub-lineage. Therefore, the classifier may select them as important as they may improve the classification for a large number of isolates. Hence, ranking them as important could be due to limitations of machine learning based feature selection (due to points explained above).

SPCA/SNMF can avoid the curse of dimensionality while keeping most variance in the data, hence leading to similar or higher performance (up to 1%, Supplementary Material H). Here, results considering 100 components were reported for all drugs (check Supplementary Material J for 50 and 150 components). We found that the dimension reduction step improved the performance of ensemble methods especially GBT for AK, MOX, OFX and KAN drugs while basic machine learning methods could not perform well for them. The trees cannot sum the effect of multiple variables and work considering one variable at a time. They run out of data instances before taking all necessary variables into account. Hence, SPCA/SNMF and similar methods can be helpful since they aggregate the information from multiple variables. Dimensionality reduction can also serve as regularization in order to prevent over-fitting. In addition, for less studied drugs the available catalog of resistance-associated mutations has not been studied completely. Hence, considering all available variants and allowing machine learning methods to reduce the dimension can improve the performance as seen in our results for CAP and AK. In terms of computational cost, all dimension reduction stages impose some time complexity to factorize the data. If we note *n* as the number of isolates and *m* as the number of features (variants), $t_{\text{max}}=\text{max}(m,n)$, and $t_{\text{min}}=\text{min}(m,n)$, then the time complexity of PCA is O($t{\text{max}}^{2}t\text{min}$) and SPCA is O(*mnk*) for extracting top *K* components. Similar time complexity can also be seen for NMF and SNMF. However, this time will be compensated later by passing fewer features to the classifiers to learn the susceptibility/resistance. Here, we did not optimize the number of SPCA/SNMF components for each drug as the feature space (eigen vectors). Optimizing the number of components could improve the performance for each drug further. However, such techniques have a limitation that they cannot be used for feature selection/mutation identification.

Finally, we note that there are several limitations regarding our analysis including the error in phenotypes, assumption of resistance independence for each drug (labels learned independently), independence of feature space for classification, considering all variants to have the same importance, ignoring missing labels and considering null calls in an isolate as no variant. Moreover, there might be some lineage related mutations as well as frequently appeared mutations ranking as important. LR-L2 (without the dimension reduction stage) or GBT (by adding it) could be two possible classifiers to be considered in the real practice or for the study of any drugs that was not reported here.

## 5 Conclusion

Several machine learning classifiers were investigated for TB resistance classification. Developed techniques were able to improve the classification of resistance from genetic data and show potential in the analysis of a large dataset with high dimensionality. Three feature spaces were considered, in which F1/SPCA-F1 for AUC and sensitivity and F3 for F1-score were more informative. The best performing classifier outperformed the assessed DA method in terms of F1-Score, AUC and sensitivity. We also showed the dimension reduction step can improve the performance of resistance classification for some drugs. Consequently, this work shows that machine learning methods can perform well considering a large number of isolates and genetic variations and results are more promising for less studied drugs. Analyzing importance of variants using machine learning techniques also shows the possibility of finding new drug-associated mutations. Considering the whole genome sequences including positions outside 23 genes and deep networks for non-linear classification and dimension reduction and also optimizing the number of SPCA/SNMF components can be considered as the future work.

## Members of the CRyPTIC consortium

Derrick W Crook, Timothy EA Peto, A Sarah Walker, Sarah J Hoosdally, Ana L Gibertoni Cruz, Joshua Carter, Clara Grazian, Samaneh Kouchaki, Yang Yang, Timothy M Walker, Philip W Fowler, Daniel Wilson and David A Clifton, University of Oxford; Zamin Iqbal and Martin Hunt, European Bioinformatics Institute; E Grace Smith, Priti Rathod, Lisa Jarrett and Daniela Matias, Public Health England, Birmingham; Daniela M Cirillo, Emanuele Borroni, Simone Battaglia, Arash Ghodousi, Andrea Spitaleri and Andrea Cabibbe, Emerging Bacterial Pathogens Unit, IRCCS San Raffaele Scientific Institute, Milan; Sabira Tahseen, National Tuberculosis Control Program Pakistan, Islamabad; Kayzad Nilgiriwala and Sanchi Shah, The Foundation for Medical Research, Mumbai; Camilla Rodrigues, Priti Kambli, Utkarsha Surve and Rukhsar Khot, P.D. Hinduja National Hospital and Medical Research Centre, Mumbai; Stefan Niemann, Thomas Kohl and Matthias Merker, Research Center Borstel; Harald Hoffmann, Nikolay Molodtsov and Sara Plesnik, Institute of Microbiology & Laboratory Medicine, IML red, Gauting; Nazir Ismail, Shaheed Vally Omar, Lavania Joseph and Elliott Marubini, National Institute for Communicable Diseases, Johannesburg; Guy Thwaites, Thuong Nguyen Thuy Thuong, Nhung Hoang Ngoc and Vijay Srinivasan, Oxford University Clinical Research Unit, Ho Chi Minh City; David Moore, Jorge Coronel and Walter Solano, London School of Hygiene and Tropical Medicine and Universidad Peruana Cayetano Heredá, Lima; George F Gao, Guangxue He, Yanlin Zhao, Aijing Ma and Chunfa Liu, China CDC, Beijing; Baoli Zhu, Institute of Microbiology, CAS, Beijing; Ian Laurenson and Pauline Claxton, Scottish Mycobacteria Reference Laboratory, Edinburgh; Robert J Wilkinson, University of Cape Town; Ajit Lalvani, Imperial College London; James Posey, CDC Atlanta; Jennifer Gardy, University of British Columbia; Jim Werngren, Public Health Agency of Sweden; Nicholas Paton, National University of Singapore; Ruwen Jou, Mei-Hua Wu, Wan-Hsuan Lin, CDC Taiwan; Lucilaine Ferrazoli, Rosaline Siqueira de Oliveira, Institute Adolfo Lutz, Sao Paolo. Authors contributing to the CRyPTIC consortium are (in alphabetical order): Irena Arandjelovic, Iñaki Comas, Francis Drobniewski, Qian Gao, Vitali Sintchenko, Philip Supply and Dick van Soolingen.

## Supplementary Material

bty949_Supplementary_Data
