# Supplementary material for: Application of machine learning techniques to tuberculosis drug resistance analysis
Source: Bioinformatics. 2018 Nov 21;35(13):2276–82. doi: 10.1093/bioinformatics/bty949 (PMC6596891; doi:10.1093/bioinformatics/bty949)
Supplement: bty949_Supplementary_Data [file bty949_supplementary_data.pdf]

Application of machine learning techniques to tuberculosis drug resistance analysis

Samaneh Kouchaki, Yang Yang, Timothy M. Walker, A. Sarah Walker, Daniel J. Wilson, Timothy E.A. Peto, Derrick W. Crook, David A. Clifton, and CRyPTIC consortium

Abbreviations

Amikacin (AK)
Area under curve (AUC)
Capreomycin (CAP)
Ciprofloxacin (CIP)
Cross validation (CV)
Direct association (DA)
Ethambutol (EMB)
Fluoroquinolones-ofloxacin (OFX)
Gradient tree boosting (GBT)
Isoniazid (INH)
Kanamycin (KAN)
Logistic regression (LR)
Multidrug-resistant TB (MDR-TB)
Moxifloxacin (MOX)
Mycobacterium tuberculosis (MTB)
Non-negative matrix factorisation (NMF)
Principal components analysis (PCA)
product-of-marginals (PM)
Pyrazinamide (PZA)
Radial basis function (RBF)
Random forest (RF)
Rifampicin (RIF)
Singular value decomposition (SVD)
Single nucleotide polymorphisms (SNPs)
Sparse non-negative matrix factorisation (SNMF)
Sparse principal components analysis (SPCA)
Streptomycin (SM)
Support vector machine (SVM)
Tuberculosis (TB)
Whole genome sequencing (WGS)

Supplementary A

Table 1. Classification methods, their parameter settings, and pros and cons.

Method	Summary	Parameter setting	Pros and cons
LR	LR is a linear model that optimises a set of weights for each feature that lead to the best classification performance. Adding a regularisation term to penalise large values in the weight vector can prevent overfitting.	This model was performed using LIBLINEAR library and L1 or L2 regularisation (LR-L1 and LR-L2).	LR is easy to implement and efficient to train and provides probabilities for outcomes. However LR cannot solve non-linear problems due to its linear decision surface and has high bias.
SVM	SVM is a binary classifier that works based on finding the widest hyperplane margin to separate samples in the feature space. The hyperplane is optimised by maximising its distance to closest training points of each class, defined as support vectors. The data is not always linearly separable and hence a kernel function may transfer the data into a linearly separable space and improve the performance.	This model was run using LIBSVM. Radial basis function (SVM-RBF) and linear (SVM-Linear) kernels were considered in this work in which Optunitiy package was used to optimise kernel parameters.	SVM has a convex optimisation and also can work for both linear and non-linear separable data. It also can handle high dimensional data. However, it needs enough samples from both positive and negative classes and it is susceptible to overfitting. Furthermore, it needs a lot of memory and CPU time.
PM	PM is a generative model and is based on class conditional independence of variables. Although its assumptions do not usually hold, PM often performs well. Regarding TB analysis, the PM method calculates the probability that an isolate has a given SNP. The prediction for a new data point is then calculated from the probability of each class label, given a new example and the training data.	A Beta(1,0.25) prior for the resistant class and a Beta(0.25,1) prior for the susceptible class were considered.	PM is fast to predict labels and can work with high dimensional data. Nevertheless if there is a category with no training data, the model will assign a zero probability (unable to predict it). Its other limitation is the independence assumption.
RF	RF is an averaging method that is based on building several independent classifiers. This model fits a number of decision tree (DT) classifiers on different subsets of the dataset and average results to produce final predictions and improve the performance.	100 estimators were considered for RF training.	It is based on training of several independent trees that can be fit in parallel. Moreover, RF usually reduce the variance. However, it needs heavier computational resources.
Adaboost	Adaboost is a boosting method based on sequentially building weak estimators (models that are only slightly better than the random prediction, i.e. small DTs). Initially, all data samples have the same weight. However, at successive iterations, the weights of mislabelled training samples by the boosted model at the previous step are increased, while the weights are decreased for the remaining training samples. Hence, as iterations proceed, the influence of difficult samples is increased. Later, it combines the predictions through a weighted majority vote with the aim of reducing the bias of the combined classifier and producing a powerful ensemble.	DT used as the base classifier and 100 estimators were considered for the training.	It is not subject to overfitting but is sensitive to noisy data and outliers. Moreover, due to sequentially building of trees, Adaboost cannot be parallelized.
GBT	GBT is a generalisation of boosting to an arbitrary differentiable loss function.	DT used as the base classifier and Binomial deviance and 100 estimators were considered for the training.	It is more robust to outliers and has high predictive power. Nonetheless, similar to Adaboost and due to the sequential nature, it cannot be parallelised.

Supplementary B

Table 2. Comparing the performance of DA and various machine learning techniques and the dimension reduction stage for INH and F1-F3.

	F1					F2					F3				
Method	Accuracy	Sensitivity	Specificity	AUC	F1-score	Accuracy	Sensitivity	Specificity	AUC	F1-Score	Accuracy	Sensitivity	Specificity	AUC	F1-Score
DA	96.65 ± 0.34	91.95 ± 1.04	98.71 ± 0.22	94.95 ± 0.54	93.72 ± 0.66	96.65 ± 0.34	91.95 ± 1.04	98.71 ± 0.22	94.95 ± 0.54	93.72 ± 0.66	96.65 ± 0.34	91.95 ± 1.04	98.71 ± 0.22	94.95 ± 0.54	93.72 ± 0.66
SVM-RBF	96.37 ± 0.29	91.17 ± 1.11	98.30 ± 0.30	97.26 ± 0.38	93.24 ± 0.55	92.89 ± 13.38	87.16 ± 1.13	98.32 ± 1.41	95.19 ± 0.60	89.59 ± 9.64	95.87 ± 0.49	87.83 ± 1.94	98.90 ± 0.27	**96.50 ± 0.43**	92.09 ± 1.02
SVM-Linear	96.37 ± 0.42	92.68 ± 0.94	97.56 ± 0.38	97.23 ± 0.43	93.33 ± 0.74	96.35 ± 0.33	89.99 ± 1.10	98.76 ± 0.23	94.68 ± 0.43	93.12 ± 0.66	96.51 ± 0.40	90.98 ± 1.32	98.60 ± 0.26	95.29 ± 0.59	**93.47 ± 0.78**
PM	91.10 ± 2.20	82.41 ± 3.28	95.43 ± 0.95	94.56 ± 0.69	83.73 ± 2.88	96.37 ± 0.29	90.14 ± 0.87	98.73 ± 0.24	**95.25 ± 0.45**	93.16 ± 0.55	96.16 ± 0.31	90.82 ± 0.99	98.18 ± 0.38	96.12 ± 0.44	92.85 ± 0.58
LR-L1	96.62 ± 0.31	92.54 ± 1.02	98.17 ± 0.31	97.61 ± 0.39	93.76 ± 0.59	96.32 ± 0.38	89.86 ± 1.16	98.75 ± 0.26	94.72 ± 0.60	93.04 ± 0.74	96.37 ± 0.34	90.13 ± 1.02	98.73 ± 0.22	95.11 ± 0.54	93.16 ± 0.66
LR-L2	96.68 ± 0.30	92.19 ± 0.94	98.38 ± 0.29	**97.89 ± 0.38**	**93.84 ± 0.56**	96.27 ± 0.37	89.65 ± 1.18	98.77 ± 0.22	94.91 ± 0.51	92.95 ± 0.73	96.29 ± 0.28	89.72 ± 0.93	98.79 ± 0.22	95.81 ± 0.45	93.00 ± 0.55
RF	96.01 ± 0.41	92.72 ± 1.08	97.26 ± 0.64	97.62 ± 0.32	92.74 ± 0.70	96.41 ± 0.36	90.24 ± 1.10	98.74 ± 0.25	94.91 ± 0.52	**93.23 ± 0.69**	96.46 ± 0.30	90.70 ± 0.90	98.64 ± 0.25	95.62 ± 0.51	93.36 ± 0.59
Adaboost	96.23 ± 0.33	90.84 ± 0.94	98.31 ± 0.25	96.77 ± 0.37	93.01 ± 0.62	96.08 ± 0.33	88.68 ± 1.16	98.88 ± 0.22	94.27 ± 0.49	92.54 ± 0.66	96.06 ± 0.33	88.63 ± 1.14	98.86 ± 0.25	94.24 ± 0.56	92.50 ± 0.67
GBT	96.59 ± 0.39	91.77 ± 1.03	98.41 ± 0.32	97.26 ± 0.41	93.65 ± 0.74	96.33 ± 0.34	89.79 ± 1.02	98.80 ± 0.24	94.90 ± 0.50	93.07 ± 0.66	96.26 ± 0.35	89.38 ± 1.11	98.86 ± 0.24	95.29 ± 0.50	92.91 ± 0.69

	SNMF					SPCA				
	SNMF-F1					SPCA-F1				
Method	Accuracy	Sensitivity	Specificity	AUC	F1-Score	Accuracy	Sensitivity	Specificity	AUC	F1-Score
SVM-RBF	94.94 ± 2.46	87.43 ± 2.62	97.77 ± 2.70	96.61 ± 1.07	90.55 ± 4.12	95.96 ± 0.39	91.63 ± 1.28	97.60 ± 0.77	96.87 ± 0.36	92.56 ± 0.66
SVM-Linear	95.92 ± 0.34	88.38 ± 1.13	98.77 ± 0.23	97.08 ± 0.41	92.38 ± 0.68	96.11 ± 0.41	90.94 ± 1.38	98.07 ± 0.59	96.70 ± 0.49	92.77 ± 0.76
PM	87.12 ± 0.55	81.91 ± 1.72	89.09 ± 0.78	92.04 ± 0.64	77.72 ± 0.93	77.22 ± 4.52	69.38 ± 4.10	80.18 ± 7.49	84.47 ± 1.07	62.97 ± 4.32
LR-L1	95.66 ± 0.36	86.70 ± 1.27	99.04 ± 0.21	96.00 ± 0.49	91.63 ± 0.74	95.77 ± 0.39	87.31 ± 1.21	98.96 ± 0.23	96.22 ± 0.50	91.87 ± 0.77
LR-L2	85.79 ± 0.63	74.89 ± 1.45	89.91 ± 0.74	91.26 ± 0.80	74.30 ± 1.08	92.42 ± 0.40	75.69 ± 1.39	98.74 ± 0.22	96.52 ± 0.39	84.55 ± 0.91
RF	95.58 ± 0.78	92.46 ± 1.34	96.79 ± 1.14	97.25 ± 0.36	92.01 ± 1.22	95.48 ± 0.68	92.02 ± 1.25	97.66 ± 0.46	97.12 ± 0.37	91.79 ± 1.10
Adaboost	96.04 ± 0.44	92.96 ± 1.04	97.21 ± 0.53	97.55 ± 0.40	92.80 ± 0.79	96.32 ± 0.35	91.64 ± 1.05	98.09 ± 0.35	97.20 ± 0.38	**93.18 ± 0.66**
GBT	96.25 ± 0.33	92.18 ± 0.98	97.79 ± 0.42	**97.65 ± 0.35**	**93.10 ± 0.63**	96.30 ± 0.34	91.23 ± 0.89	98.21 ± 0.34	**97.21 ± 0.38**	93.11 ± 0.57

Table 3. Comparing the performance of DA and various machine learning techniques and the dimension reduction stage for EMB and F1-F3.

	F1					F2					F3				
Method	Accuracy	Sensitivity	Specificity	AUC	F1-Score	Accuracy	Sensitivity	Specificity	AUC	F1-Score	Accuracy	Sensitivity	Specificity	AUC	F1-Score
DA	93.64 ± 0.41	83.31 ± 1.62	95.17 ± 0.38	89.24 ± 0.85	77.16 ± 1.38	93.64 ± 0.41	83.31 ± 1.62	95.17 ± 0.38	89.24 ± 0.85	77.16 ± 1.38	93.64 ± 0.41	83.31 ± 1.62	95.17 ± 0.38	89.24 ± 0.85	77.16 ± 1.38
SVM-RBF	91.36 ± 0.84	93.08 ± 1.62	91.10 ± 1.07	95.80 ± 0.49	73.57 ± 1.84	86.98 ± 2.85	80.71 ± 1.34	87.91 ± 5.93	88.68 ± 5.60	71.28 ± 4.53	93.75 ± 0.45	88.05 ± 2.24	94.57 ± 0.45	94.42 ± 0.71	78.34 ± 1.49
SVM-Linear	92.05 ± 0.89	91.32 ± 1.71	92.16 ± 1.07	95.72 ± 0.64	74.82 ± 2.09	93.61 ± 0.45	83.00 ± 1.84	95.18 ± 0.40	89.32 ± 0.92	77.01 ± 1.53	93.66 ± 0.50	89.33 ± 1.76	94.30 ± 0.62	94.39 ± 0.74	78.43 ± 1.40
PM	90.52 ± 1.94	88.67 ± 1.82	90.79 ± 2.27	94.18 ± 0.75	70.89 ± 3.72	93.63 ± 0.35	83.25 ± 1.74	95.17 ± 0.34	89.77 ± 0.94	77.12 ± 1.21	91.41 ± 3.75	83.66 ± 3.65	92.56 ± 4.74	92.73 ± 0.89	72.52 ± 6.84
LR-L1	92.19 ± 0.81	91.86 ± 1.63	92.33 ± 0.98	95.97 ± 0.62	**75.23 ± 1.96**	93.63 ± 0.39	83.21 ± 1.61	95.17 ± 0.40	**89.80 ± 0.87**	**77.10 ± 1.26**	93.94 ± 0.45	89.18 ± 1.63	94.64 ± 0.45	95.21 ± 0.55	**79.14 ± 1.37**
LR-L2	91.92 ± 0.63	92.12 ± 1.84	91.89 ± 0.84	**96.25 ± 0.54**	74.65 ± 1.46	93.62 ± 0.39	83.15 ± 1.64	95.17 ± 0.37	89.77 ± 0.88	77.06 ± 1.33	93.81 ± 0.37	89.04 ± 1.65	94.52 ± 0.37	**95.38 ± 0.59**	78.77 ± 1.18
RF	90.08 ± 1.13	91.79 ± 2.06	89.82 ± 1.50	95.31 ± 0.47	70.54 ± 2.17	93.56 ± 0.38	81.59 ± 2.76	95.33 ± 0.43	89.71 ± 1.17	76.55 ± 1.44	93.39 ± 0.55	87.40 ± 1.61	94.97 ± 0.55	94.97 ± 0.55	77.34 ± 1.59
Adaboost	89.01 ± 0.51	88.92 ± 1.93	89.03 ± 0.60	94.37 ± 0.64	67.61 ± 1.21	93.56 ± 0.43	79.12 ± 2.15	95.55 ± 0.40	88.50 ± 0.97	75.64 ± 1.32	92.96 ± 2.14	80.69 ± 2.95	94.77 ± 0.27	92.78 ±0.90	75.07 ± 4.11
GBT	90.28 ± 1.06	92.68 ± 1.87	89.93 ± 1.35	95.49 ± 0.53	71.16 ± 2.16	93.55 ± 0.43	82.06 ± 2.37	95.26 ± 0.45	89.77 ± 1.07	76.66 ± 1.51	94.00 ± 0.45	87.05 ± 1.87	95.03 ± 0.44	94.86 ± 0.58	78.92 ± 1.45

	SNMF					SPCA				
	SNMF-F1					SPCA-F1				
Method	Accuracy	Sensitivity	Specificity	AUC	F1-Score	Accuracy	Sensitivity	Specificity	AUC	F1-Score
SVM-RBF	90.99 ± 0.98	89.60 ± 1.92	91.96 ± 1.18	94.78 ± 0.74	71.99 ± 2.05	83.44 ± 3.76	90.18 ± 5.93	82.44 ± 7.81	89.18 ± 2.41	68.07 ± 5.85
SVM-Linear	91.36 ± 0.57	90.53 ± 1.74	91.48 ± 0.67	95.03 ± 0.65	**72.99 ± 1.39**	92.02 ± 0.58	89.65 ± 2.01	92.38 ± 0.79	**95.62 ± 0.51**	74.37 ± 1.36
PM	85.38 ± 1.94	81.15 ± 2.47	85.99 ± 2.40	90.57 ± 0.98	59.00 ± 2.90	84.48 ± 5.30	73.49 ± 3.80	86.10 ± 6.42	86.95 ± 1.28	56.25 ± 7.92
LR-L1	90.64 ± 0.91	88.88 ± 2.36	90.90 ± 1.18	94.49 ± 0.64	71.05 ± 1.97	90.29 ± 1.39	86.89 ± 2.94	90.79 ± 1.88	93.99 ± 0.66	69.89 ± 2.71
LR-L2	84.30 ± 2.92	83.75 ± 2.21	90.93 ± 0.63	90.93 ± 0.63	57.90 ± 1.39	92.47 ± 0.49	86.80 ± 1.67	93.30 ± 0.55	95.57 ± 0.48	**74.82 ± 1.38**
RF	88.99 ± 1.04	90.50 ± 1.94	88.77 ± 1.30	94.57 ± 0.64	68.00 ± 1.97	90.48 ± 0.75	88.95 ± 2.37	90.71 ± 0.99	94.75 ± 0.57	70.69 ± 1.61
Adaboost	89.27 ± 0.67	93.69 ± 1.29	88.61 ± 0.78	95.31 ± 0.55	69.25 ± 1.40	89.60 ± 0.52	92.40 ± 1.45	89.18 ± 0.63	94.97 ± 0.52	69.60 ± 1.14
GBT	90.83 ± 0.86	92.15 ± 1.76	90.64 ± 1.06	**95.70 ± 0.52**	72.19 ± 1.90	90.68 ± 0.77	92.30 ± 1.70	90.44 ± 0.94	95.58 ± 0.61	71.89 ± 1.70

Table 4. Comparing the performance of DA and various machine learning techniques and the dimension reduction stage for RIF and F1-F3.

	F1					F2					F3				
Method	Accuracy	Sensitivity	Specificity	AUC	F1-Score	Accuracy	Sensitivity	Specificity	AUC	F1-Score	Accuracy	Sensitivity	Specificity	AUC	F1-Score
DA	97.16 ± 0.30	91.70 ± 1.19	98.73 ± 0.22	95.22 ± 0.59	93.52 ± 0.71	97.16 ± 0.30	91.70 ± 1.19	98.73 ± 0.22	95.22 ± 0.59	93.52 ± 0.71	97.16 ± 0.30	91.70 ± 1.19	98.73 ± 0.22	95.22 ± 0.59	93.52 ± 0.71
SVM-RBF	96.31 ± 0.70	93.03 ± 1.02	97.26 ± 0.99	97.89 ± 0.38	91.89 ± 1.40	87.13 ± 3.18	88.51 ± 2.40	87.45 ± 2.06	95.59 ± 0.55	84.60 ± 1.22	96.94 ± 0.37	90.36 ± 1.18	98.84 ± 0.28	96.00 ± 0.77	92.96 ± 0.91
SVM-Linear	96.67 ± 0.67	92.50 ± 0.89	97.87 ± 0.85	97.82 ± 0.40	92.56 ± 1.35	97.11 ± 0.23	98.77 ± 0.20	95.53 ± 0.70	95.12 ± 0.45	93.40 ± 0.53	97.13 ± 0.31	91.50 ± 1.10	98.76 ± 0.28	96.43 ± 0.73	93.46 ± 0.71
PM	92.19 ± 2.56	87.79 ± 2.10	93.45 ± 3.72	95.86 ± 0.51	83.68 ± 4.05	97.10 ± 0.33	98.76 ± 0.21	95.51 ± 0.74	**95.64 ± 0.58**	**93.37 ± 0.77**	96.95 ± 0.31	91.00 ± 1.34	98.66 ± 0.24	96.65 ± 0.51	93.02 ± 0.73
LR-L1	96.69 ± 0.49	92.27 ± 1.25	97.96 ± 0.77	97.96 ± 0.77	**92.58 ± 1.01**	97.08 ± 0.27	91.21 ± 0.89	98.77 ± 0.23	95.45 ± 0.47	93.33 ± 0.63	97.10 ± 0.26	91.04 ± 1.07	98.84 ± 0.22	96.50 ± 0.44	93.35 ± 0.62
LR-L2	96.40 ± 0.42	92.77 ± 1.28	97.45 ± 0.63	**98.08 ± 0.32**	92.04 ± 0.88	97.03 ± 0.28	90.93 ± 1.02	98.79 ± 0.22	95.53 ± 0.52	93.20 ± 0.66	97.09 ± 0.34	90.92 ± 1.20	98.87 ± 0.25	**96.78 ± 0.53**	93.32 ± 0.80
RF	95.15 ± 0.60	92.66 ± 1.18	95.87 ± 0.88	97.67 ± 0.42	89.54 ± 1.15	97.07 ± 0.33	90.80 ± 1.35	98.88 ± 0.23	95.50 ± 0.58	93.27 ± 0.78	97.17 ± 0.29	91.72 ± 1.10	99.43 ±0.22	96.60 ± 0.51	**93.53 ± 0.68**
Adaboost	94.10 ± 0.51	92.41 ± 1.34	94.59 ± 0.59	97.03 ± 0.46	87.52 ± 1.02	95.90 ± 0.33	83.61 ± 1.51	99.45 ± 0.20	92.30 ± 0.74	90.13 ± 0.87	95.91 ± 0.40	83.73 ± 1.97	99.43 ± 0.22	93.36 ± 0.75	90.16 ± 1.07
GBT	95.40 ± 0.76	92.39 ± 1.34	96.27 ± 1.23	97.71 ± 0.35	90.02 ± 1.44	96.94 ± 0.30	89.90 ± 1.33	98.97 ± 0.18	95.41 ± 0.58	92.93 ± 0.73	96.98 ± 0.25	89.99 ± 1.19	98.99 ± 0.26	96.24 ± 0.40	93.02 ± 0.60

	SNMF					SPCA				
	SNMF-F1					SPCA-F1				
Method	Accuracy	Sensitivity	Specificity	AUC	F1-Score	Accuracy	Sensitivity	Specificity	AUC	F1-Score
SVM-RBF	92.30 ± 1.27	87.12 ± 2.77	93.80 ± 1.86	96.66 ± 0.71	83.56 ± 2.18	95.44 ± 0.45	90.64 ± 1.36	96.82 ± 0.63	97.37 ± 0.39	89.89 ± 0.93
SVM-Linear	87.39 ± 1.94	87.07 ± 3.72	87.49 ± 3.45	94.73 ± 0.49	75.71 ± 2.55	95.62 ± 0.45	90.57 ± 1.17	97.08 ± 0.56	97.32 ± 0.41	**90.26 ± 0.96**
PM	87.34 ± 1.43	84.59 ± 2.25	88.13 ± 2.19	93.39 ± 0.68	75.01 ± 1.95	85.46 ± 6.21	73.57 ± 3.76	88.88 ± 8.95	87.85 ± 1.16	70.60 ± 7.79
LR-L1	94.04 ± 0.42	88.42 ± 1.41	95.66 ± 0.46	96.77 ± 0.51	86.90 ± 0.91	94.32 ± 0.90	89.59 ± 1.89	95.69 ± 1.55	96.59 ± 0.53	87.64 ± 1.62
LR-L2	85.53 ± 0.76	77.61 ± 1.60	87.81 ± 0.85	90.96 ± 0.82	86.90 ± 0.91	94.75 ± 0.44	82.95 ± 1.54	98.15 ± 0.27	97.10 ± 0.47	87.60 ± 1.08
RF	94.64 ± 0.94	92.06 ± 1.52	95.38 ± 1.48	97.39 ± 0.36	88.53 ± 1.71	95.65 ± 0.49	91.15 ± 1.15	97.66 ± 0.46	97.28 ±0.39	90.37 ± 0.99
Adaboost	95.38 ± 0.55	92.98 ± 1.02	96.07 ± 0.76	97.68 ± 0.41	90.02 ± 1.08	94.61 ± 0.46	93.05 ± 0.97	98.09 ± 0.35	**97.63 ± 0.35**	88.55 ± 0.92
GBT	95.92 ± 0.53	92.30 ± 1.43	96.69 ± 0.77	**97.79 ± 0.39**	**91.02 ± 1.07**	95.63 ± 0.59	91.63 ± 1.48	98.21 ± 0.34	97.63 ± 0.98	90.37 ± 1.12

Table 5. Comparing the performance of DA and various machine learning techniques and the dimension reduction stage for PZA and F1-F3.

	F1					F2					F3				
Method	Accuracy	Sensitivity	Specificity	AUC	F1-Score	Accuracy	Sensitivity	Specificity	AUC	F1-Score	Accuracy	Sensitivity	Specificity	AUC	F1-Score
DA	92.15 ± 0.36	43.11 ± 2.97	98.46 ± 0.27	70.78 ± 1.46	55.55 ± 2.68	92.15 ± 0.36	43.11 ± 2.97	98.46 ± 0.27	70.78 ± 1.46	55.55 ± 2.68	92.15 ± 0.36	43.11 ± 2.97	98.46 ± 0.27	70.78 ± 1.46	55.55 ± 2.68
SVM-RBF	88.80 ± 1.68	88.05 ± 2.53	88.90 ± 2.08	93.32 ± 0.86	64.39 ± 3.25	77.62 ± 3.03	52.91 ± 5.17	80.80 ± 2.12	68.43 ± 3.90	49.01 ± 3.54	88.95 ± 3.70	76.13 ± 5.43	90.59 ± 4.77	**88.69 ± 1.64**	62.06 ± 6.06
SVM-Linear	89.46 ± 1.52	87.01 ± 3.01	89.77 ± 1.92	93.11 ± 0.85	**65.46 ± 2.98**	90.45 ± 1.30	41.39 ± 4.85	96.76 ± 1.83	69.40 ± 1.51	52.88 ± 5.47	87.32 ± 3.40	73.68 ± 5.42	89.07 ± 4.40	85.15 ± 2.38	57.92 ± 6.58
PM	88.16 ± 1.30	84.77 ± 2.69	88.60 ± 1.84	92.02 ± 1.05	62.14 ± 2.72	92.05 ± 0.43	39.79 ± 2.77	98.78 ± 0.28	68.04 ± 1.55	**53.28 ± 2.94**	84.34 ± 0.98	77.39 ± 2.30	85.23 ± 1.15	83.47 ± 1.52	53.02 ± 1.72
LR-L1	89.25 ± 1.30	87.23 ± 3.04	89.51 ± 1.70	93.45 ± 0.90	65.03 ± 2.44	91.81 ± 0.42	34.92 ± 3.27	99.13 ± 2.43	68.12 ± 1.45	49.21 ± 3.58	84.21 ± 0.94	70.83 ± 2.99	85.94 ± 1.15	83.40 ±1.30	50.60 ± 1.75
LR-L2	88.82 ± 1.29	88.12 ± 2.65	88.91 ± 1.66	**93.89 ± 0.80**	64.36 ± 2.41	92.08 ± 0.43	39.59 ± 3.22	98.84 ± 0.29	**69.59 ± 1.61**	53.21 ± 3.21	84.32 ± 0.85	77.58 ± 2.64	85.19 ± 0.88	85.43 ± 1.38	53.03 ± 1.86
RF	87.27 ± 1.57	87.60 ± 2.90	87.23 ± 1.96	93.18 ± 0.97	61.22 ± 2.74	92.06 ± 0.46	39.13 ± 3.07	98.87 ± 0.27	69.53 ± 1.46	52.86 ± 3.30	90.50 ± 3.79	65.35 ± 7.74	93.74 ± 5.16	86.17 ± 1.47	**62.49 ± 6.99**
Adaboost	86.18 ± 0.67	85.17 ± 2.90	86.31 ± 0.69	91.63 ± 0.98	58.43 ± 1.74	90.48 ± 0.23	18.13 ± 2.05	99.79 ± 0.09	58.96 ± 1.01	30.21 ± 2.87	74.43 ± 21.23	56.23 ± 17.68	76.77 ± 26.20	71.74 ± 1.53	37.31 ± 6.34
GBT	87.36 ± 1.46	88.49 ± 2.76	87.22 ± 1.86	92.82 ± 0.98	61.61 ± 2.54	91.93 ± 0.32	35.89 ± 2.52	99.14 ± 0.21	68.07 ± 1.29	50.31 ± 2.64	84.69 ± 0.98	64.61 ± 3.41	87.27 ± 1.09	81.08 ± 1.38	49.06 ± 2.27

	SNMF					SPCA				
	SNMF-F1					SPCA-F1				
Method	Accuracy	Sensitivity	Specificity	AUC	F1-Score	Accuracy	Sensitivity	Specificity	AUC	F1-Score
SVM-RBF	84.90 ± 2.92	80.38 ± 5.88	85.49 ± 3.13	90.03 ± 2.40	55.16 ± 5.52	87.55 ± 11.01	85.76 ± 4.92	87.78 ± 12.73	92.35 ± 1.05	63.39 ± 6.73
SVM-Linear	86.65 ± 1.48	81.37 ± 3.49	87.33 ± 1.96	91.46 ± 0.83	58.28 ± 2.49	89.16 ± 0.61	86.58 ± 2.20	89.50 ± 0.78	92.23 ± 0.99	**64.58 ± 1.37**
PM	85.45 ± 1.30	82.63 ± 2.53	85.81 ± 1.57	88.84 ± 1.17	56.51 ± 2.19	86.49 ± 0.94	78.86 ± 3.05	87.48 ± 1.06	90.06 ± 1.04	57.14 ± 2.11
LR-L1	86.44 ± 0.66	81.05 ± 2.37	87.13 ± 0.71	88.75 ± 1.43	57.69 ± 1.55	86.51 ± 0.85	85.57 ± 1.80	86.63 ± 0.98	90.54 ± 0.97	59.16 ± 1.69
LR-L2	81.39 ± 1.10	76.32 ± 4.37	82.04 ± 1.30	85.82 ± 1.14	48.33 ± 2.15	89.70 ± 0.51	81.96 ± 2.35	90.70 ± 0.56	92.30 ± 0.78	64.48 ± 1.48
RF	85.81 ± 1.73	87.01 ± 3.06	85.66 ± 2.13	92.61 ± 0.78	58.45 ± 2.82	87.93 ± 1.45	83.61 ± 3.38	88.49 ± 1.89	91.80 ± 0.87	61.37 ± 2.55
Adaboost	85.95 ± 1.70	89.94 ± 2.08	85.44 ± 2.03	92.98 ± 0.94	59.48 ± 2.71	86.90 ± 1.09	88.02 ± 2.04	86.75 ± 1.19	92.50 ± 0.96	60.56 ± 2.17
GBT	88.32 ± 1.11	88.14 ± 2.46	88.34 ± 1.36	**93.50 ± 0.82**	**63.32 ± 2.18**	88.37 ± 1.38	86.82 ± 2.66	88.57 ± 1.78	**92.94 ± 0.72**	63.12 ± 2.46

Table 6. Comparing the performance of DA and various machine learning techniques and the dimension reduction stage for SM and F1-F3.

	F1					F2					F3				
Method	Accuracy	Sensitivity	Specificity	AUC	F1-Score	Accuracy	Sensitivity	Specificity	AUC	F1-Score	Accuracy	Sensitivity	Specificity	AUC	F1-Score
DA	93.41 ± 0.62	82.80 ± 1.90	97.19 ± 0.44	89.99 ± 0.99	86.83 ± 1.29	93.41 ± 0.62	82.80 ± 1.90	97.19 ± 0.44	89.99 ± 0.99	86.83 ± 1.29	93.41 ± 0.62	82.80 ± 1.90	97.19 ± 0.44	89.99 ± 0.99	86.83 ± 1.29
SVM-RBF	92.07 ± 0.72	87.20 ± 2.28	93.81 ± 1.27	95.02 ± 0.74	85.24 ± 1.86	91.30 ± 1.45	78.22 ± 1.39	95.96 ± 1.73	**90.37 ± 1.11**	83.60 ± 1.79	92.69 ± 0.98	82.38 ± 3.62	96.36 ± 1.90	**93.48 ± 1.13**	85.55 ± 1.69
SVM-Linear	92.27 ± 0.87	86.68 ± 2.44	94.26 ± 1.53	94.26 ± 0.81	85.49 ± 1.40	92.93 ± 0.59	79.45 ± 2.08	97.72 ± 0.53	89.01 ± 0.98	85.48 ± 1.30	93.29 ± 0.46	83.54 ± 1.50	96.75 ± 0.53	91.35 ± 1.06	86.71 ± 0.93
PM	84.24 ± 2.63	81.35 ± 2.38	85.26 ± 4.02	91.17 ± 0.96	73.19 ± 3.19	93.00 ± 0.68	80.10 ± 1.97	97.59 ± 0.59	88.50 ± 1.23	**85.72 ± 1.43**	92.01 ± 0.73	74.74 ± 2.79	98.15 ± 0.45	88.57 ± 1.16	83.04 ± 1.78
LR-L1	92.43 ± 0.64	86.86 ± 2.97	94.42 ± 1.42	94.67 ± 0.72	**85.76 ± 1.07**	92.81 ± 0.52	78.46 ± 1.99	97.92 ± 0.41	89.09 ± 0.90	85.13 ± 1.19	93.37 ± 0.66	81.54 ± 1.93	97.58 ± 0.63	91.84 ± 1.18	86.58 ± 1.36
LR-L2	92.24 ± 0.77	87.40 ± 1.98	94.15 ± 1.23	**95.15 ± 0.56**	85.76 ± 1.28	92.89 ± 0.56	78.92 ± 1.91	97.86 ± 0.46	89.75 ± 0.82	85.33 ± 1.24	93.41 ± 0.49	81.64 ± 1.70	97.60 ± 4.35	92.31 ± 0.83	86.66 ± 1.06
RF	91.24 ± 1.14	87.69 ± 2.44	92.50 ± 2.03	94.86 ± 0.60	84.04 ± 1.63	92.99 ± 0.58	79.73 ± 2.15	97.71 ± 0.58	89.67 ± 1.06	85.64 ± 1.28	93.61 ± 0.58	83.03 ± 1.72	97.61 ± 0.50	92.08 ± 0.92	**87.21 ± 1.19**
Adaboost	91.80 ± 0.70	86.89 ± 1.97	93.55 ± 0.69	93.26 ± 0.95	84.76 ± 1.33	90.94 ± 1.21	68.18 ± 2.42	99.03 ± 0.45	87.40 ± 1.07	79.67 ± 3.38	92.82 ± 0.76	76.88 ± 2.44	98.49 ± 0.40	88.53 ± 1.19	84.87 ± 1.74
GBT	91.90 ± 0.82	87.90 ± 1.69	93.28 ± 1.21	94.15 ± 0.81	85.08 ± 1.31	92.82 ± 0.60	78.10 ± 2.01	98.06 ± 0.39	89.53 ± 0.99	85.08 ± 1.35	93.38 ± 0.61	80.03 ± 2.21	98.13 ± 0.45	91.59 ± 0.94	86.37 ± 1.38

	SNMF					SPCA				
	SNMF-F1					SPCA-F1				
Method	Accuracy	Sensitivity	Specificity	AUC	F1-Score	Accuracy	Sensitivity	Specificity	AUC	F1-Score
SVM-RBF	88.00 ± 3.29	79.75 ± 1.72	90.93 ± 2.15	92.13 ± 1.73	77.26 ± 2.58	92.15 ± 0.70	85.24 ± 2.97	94.61 ± 0.94	94.22 ± 0.94	85.06 ± 1.46
SVM-Linear	89.73 ± 0.92	82.99 ± 2.43	92.12 ± 1.06	93.20 ± 1.01	80.91 ± 1.68	92.33 ± 0.61	86.11 ± 1.31	94.54 ± 0.73	94.28 ± 0.59	**85.48 ± 1.08**
PM	85.11 ± 1.06	82.95 ± 1.98	85.87 ± 1.62	90.32 ± 0.72	74.52 ± 1.40	75.11 ± 1.11	74.88 ± 2.32	75.19 ± 1.17	85.38 ± 1.21	61.21 ± 1.63
LR-L1	90.01 ± 0.76	82.27 ± 1.96	92.76 ± 0.76	91.40 ± 0.91	81.20 ± 1.44	91.79 ± 0.71	86.32 ± 1.94	93.74 ± 0.80	93.38 ± 0.80	84.66 ± 1.32
LR-L2	80.34 ± 2.45	66.09 ± 7.35	85.41 ± 5.64	86.45 ± 1.15	63.84 ± 1.91	91.95 ± 0.63	81.37 ± 1.82	95.72 ± 0.65	93.20 ± 0.78	84.14 ± 1.25
RF	89.76 ± 1.10	87.73 ± 2.48	90.49 ± 1.85	94.66 ± 0.60	81.83 ± 1.58	91.64 ± 0.73	85.42 ± 2.23	93.84 ± 1.30	94.28 ± 0.68	84.28 ± 1.20
Adaboost	91.10 ± 0.83	88.25 ± 1.86	92.11 ± 1.04	94.63 ± 0.68	83.88 ± 1.42	91.23 ± 0.71	88.05 ± 1.70	92.36 ± 0.83	94.28 ± 0.83	84.04 ± 1.22
GBT	91.66 ± 0.80	87.45 ± 2.14	93.16 ± 1.40	**95.09 ± 0.61**	**84.63 ± 1.25**	92.02 ± 0.66	87.04 ± 1.69	93.80 ± 0.96	**94.45 ± 0.81**	85.13 ± 1.13

Table 7. Comparing the performance of DA and various machine learning techniques and the dimension reduction stage for AK and F1-F3.

	F1					F2					F3				
Method	Accuracy	Sensitivity	Specificity	AUC	F1-Score	Accuracy	Sensitivity	Specificity	AUC	F1-Score	Accuracy	Sensitivity	Specificity	AUC	F1-Score
DA	96.52 ± 0.59	65.21 ± 5.32	97.19 ± 0.44	82.46 ± 2.70	77.46 ± 4.25	96.52 ± 0.59	65.21 ± 5.32	97.19 ± 0.44	82.46 ± 2.70	77.46 ± 4.25	96.52 ± 0.59	65.21 ± 5.32	97.19 ± 0.44	82.46 ± 2.70	77.46 ± 4.25
SVM-RBF	87.09 ± 5.57	78.53 ± 7.97	87.96 ± 6.74	90.23 ± 3.01	55.33 ± 10.66	63.32 ± 2.34	77.47 ± 8.24	61.87 ± 2.34	62.56 ± 2.41	53.94 ± 5.17	96.02 ± 1.35	65.27 ± 6.55	99.14 ± 1.41	88.41 ± 4.10	**75.32 ± 6.13**
SVM-Linear	86.16 ± 4.50	77.36 ± 7.84	87.06 ± 5.55	89.20 ± 2.38	52.06 ± 7.54	96.53 ± 0.47	64.84 ± 5.21	99.75 ± 0.20	82.26 ± 2.61	77.40 ± 3.75	94.02 ± 3.41	67.18 ± 5.91	96.74 ± 3.95	84.94 ± 4.68	69.33 ± 10.54
PM	71.30 ± 6.48	87.94 ± 5.12	69.61 ± 7.42	88.18 ± 2.49	36.77 ± 4.72	96.54 ± 0.54	64.83 ± 4.84	99.75 ± 0.22	82.13 ±2.43	77.44 ± 4.00	87.53 ± 12.64	72.62 ± 12.05	89.04 ± 14.97	89.02 ± 2.12	61.36 ± 18.03
LR-L1	91.64 ± 5.90	71.49 ± 10.78	93.69 ± 7.27	91.00 ± 2.94	**65.12 ± 12.82**	96.59 ± 0.56	64.82 ± 5.79	99.81 ± 0.14	82.32 ± 2.91	**77.66 ± 3.38**	92.96 ± 7.35	68.51 ± 8.81	95.44 ± 8.70	**89.85 ± 3.04**	69.72 ± 14.44
LR-L2	90.48 ± 6.15	73.51 ± 9.09	92.20 ± 7.53	**91.22 ± 2.35**	62.71 ± 12.90	96.52 ± 0.62	64.84 ± 5.86	99.73 ± 0.23	82.26 ± 2.94	77.30 ± 4.55	95.25 ± 4.60	67.19 ± 6.84	98.10 ± 5.42	89.73 ± 2.83	74.80 ± 9.70
RF	84.19 ± 5.30	80.09 ± 8.38	84.61 ± 6.50	89.93 ± 2.24	49.69 ± 7.11	96.52 ± 0.61	64.84 ± 6.00	99.74 ± 1.86	82.29 ± 3.01	77.33 ± 4.59	95.50 ± 3.68	65.54 ± 6.55	98.60 ± 4.23	89.24 ± 3.21	74.64 ± 8.58
Adaboost	78.31 ± 2.30	78.33 ± 14.43	78.31 ± 26.45	90.42 ± 2.59	51.96 ± 20.20	96.57 ± 0.54	64.84 ± 5.53	99.79 ± 1.73	**82.32 ± 2.76**	77.58 ± 4.17	94.49 ± 5.77	61.72 ± 10.96	97.81 ± 7.01	89.62 ± 2.44	70.72 ± 11.01
GBT	93.94 ± 4.93	73.82 ± 7.72	92.68 ± 5.97	90.57 ± 2.67	62.70 ± 11.21	96.51 ± 0.60	64.84 ± 5.89	99.72 ± 0.23	82.24 ± 2.94	77.25 ± 4.69	96.07 ± 1.17	61.86 ± 9.66	99.55 ± 1.03	89.46 ± 2.46	74.05 ± 8.90

	SNMF					SPCA				
	SNMF-F1					SPCA-F1				
Method	Accuracy	Sensitivity	Specificity	AUC	F1-Score	Accuracy	Sensitivity	Specificity	AUC	F1-Score
SVM-RBF	75.88 ± 10.57	73.96 ± 9.60	76.07 ± 12.14	82.80 ± 8.80	37.36 ± 5.90	45.61 ± 11.15	86.45 ± 16.46	41.46 ± 16.90	49.82 ± 13.30	37.39 ± 13.98
SVM-Linear	80.40 ± 4.09	73.59 ± 1.22	81.10 ± 5.14	84.60 ± 2.99	40.90 ± 6.10	86.74 ± 21.68	72.50 ± 11.25	88.18 ± 24.83	88.67 ± 12.95	63.61 ± 16.42
PM	79.02 ± 3.69	79.31 ± 5.62	79.96 ± 4.36	86.77 ± 2.08	42.51 ± 4.08	85.22 ± 4.25	80.00 ± 7.57	85.75 ± 5.09	90.50 ± 2.58	50.96 ± 7.01
LR-L1	82.48 ± 6.73	68.42 ± 9.13	83.91 ± 8.09	83.94 ± 3.00	43.80 ± 8.11	96.59 ± 0.58	64.83 ± 5.77	99.81 ± 0.16	82.32 ± 2.91	**77.66 ± 4.44**
LR-L2	73.40 ± 4.53	71.38 ± 11.05	73.60 ± 5.86	80.26 ± 2.80	33.24 ± 3.11	88.68 ± 2.48	77.23 ± 6.96	89.84 ± 3.05	**91.37 ± 2.36**	56.14 ± 5.03
RF	90.79 ± 4.23	73.42 ± 8.25	92.55 ± 5.22	**90.21 ± 2.55**	**61.33 ± 9.04**	90.87 ± 4.37	73.01 ± 8.28	90.63 ± 2.46	90.63 ± 2.46	61.52 ± 9.67
Adaboost	81.75 ± 2.48	80.04 ± 4.85	81.92 ± 2.89	88.51 ± 2.72	44.90 ± 3.36	90.53 ± 6.99	72.70 ± 1.03	92.35 ± 8.58	90.25 ± 2.72	63.26 ± 13.56
GBT	90.63 ± 4.40	71.89 ± 8.07	92.53 ± 5.47	89.52 ± 2.78	60.60 ± 9.33	90.83 ± 5.05	73.40 ± 8.07	92.60 ± 6.13	90.94 ± 2.45	62.41 ± 11.35

Table 8. Comparing the performance of DA and various machine learning techniques and the dimension reduction stage for MOX and F1-F3.

	F1					F2					F3				
Method	Accuracy	Sensitivity	Specificity	AUC	F1-Score	Accuracy	Sensitivity	Specificity	AUC	F1-Score	Accuracy	Sensitivity	Specificity	AUC	F1-Score
DA	92.59 ± 1.28	62.97 ± 6.60	98.80 ± 0.68	80.89 ± 3.32	74.49 ± 5.09	92.59 ± 1.28	62.97 ± 6.60	98.80 ± 0.68	80.89 ± 3.32	74.49 ± 5.09	92.59 ± 1.28	62.97 ± 6.60	98.80 ± 0.68	80.89 ± 3.32	74.49 ± 5.09
SVM-RBF	82.08 ± 5.32	78.12 ± 9.72	82.91 ± 7.75	87.69 ± 3.05	60.90 ± 6.14	83.57 ± 14.48	67.38 ± 13.16	86.97 ± 12.12	75.33 ± 17.32	69.16 ± 15.11	72.29 ± 13.57	75.94 ± 14.98	71.51 ± 19.99	70.55 ± 17.69	62.24 ± 19.94
SVM-Linear	83.94 ± 4.81	76.83 ± 7.79	85.55 ± 6.82	88.12 ± 3.17	63.13 ± 6.22	92.56 ± 0.94	62.82 ± 4.53	98.80 ± 0.47	80.81 ± 2.33	74.47 ± 3.75	92.41 ± 1.14	68.43 ± 6.18	97.45 ± 1.36	84.99 ± 2.81	75.69 ± 3.65
PM	64.73 ± 5.20	87.89 ± 5.16	59.86 ± 6.72	83.80 ± 3.18	46.60 ± 3.58	85.01 ± 22.61	66.34 ± 12.91	88.94 ± 29.65	80.85 ± 3.27	69.76 ± 14.38	89.34 ± 14.00	70.23 ± 8.09	93.35 ± 8.09	85.02 ± 2.16	74.28 ± 10.72
LR-L1	85.29 ± 5.55	74.39 ± 10.15	87.57 ± 8.17	89.49 ± 2.42	64.66 ± 6.73	92.48 ± 1.30	62.39 ± 6.44	98.80 ± 0.55	80.72 ± 3.44	74.06 ± 5.29	92.84 ± 1.13	65.58 ± 6.94	98.56 ± 0.71	83.41 ± 3.33	75.86 ± 4.76
LR-L2	81.12 ± 6.45	80.08 ± 9.26	81.35 ± 9.29	89.55 ± 2.84	60.66 ± 6.79	92.51 ± 1.19	62.53 ± 6.22	98.80 ± 0.48	80.88 ± 3.05	74.17 ± 4.94	92.58 ± 1.54	67.63 ± 5.14	97.81 ± 1.58	**85.56 ± 2.56**	**75.98 ± 4.50**
RF	78.10 ± 5.10	79.94 ± 7.74	77.72 ± 7.10	86.86 ± 2.74	56.37 ± 5.09	92.59 ± 1.02	62.98 ± 5.48	98.80 ± 0.51	**80.88 ± 2.72**	**74.55 ± 4.15**	92.26 ± 1.52	64.45 ± 9.27	98.60 ± 4.23	85.37 ± 3.14	73.95 ± 6.38
Adaboost	58.20 ± 9.60	90.58 ± 4.56	51.41 ± 12.02	87.26 ± 3.11	43.42 ± 4.22	84.79 ± 22.53	65.20 ± 13.40	88.90 ± 29.64	80.09 ± 3.33	68.82 ± 14.15	90.37 ± 9.83	58.44 ± 9.87	97.81 ± 7.01	81.11 ± 2.83	69.65 ± 9.33
GBT	85.39 ± 5.69	76.84 ± 9.29	87.19 ± 8.21	**90.27 ± 2.96**	**65.69 ± 7.01**	92.59 ± 1.05	62.97 ± 5.27	98.80 ± 0.60	80.85 ± 2.64	74.55 ± 4.19	92.67 ± 1.11	64.95 ± 6.74	99.55 ± 1.03	84.86 ± 2.73	75.18 ± 4.48

	SNMF					SPCA				
	SNMF-F1					SPCA-F1				
Method	Accuracy	Sensitivity	Specificity	AUC	F1-Score	Accuracy	Sensitivity	Specificity	AUC	F1-Score
SVM-RBF	69.04 ± 9.09	73.92 ± 9.68	68.02 ± 12.12	78.75 ± 7.90	45.90 ± 4.97	69.19 ± 12.18	80.95 ± 13.02	66.72 ± 17.64	74.62 ± 18.94	56.39 ± 16.13
SVM-Linear	75.20 ± 4.37	70.14 ± 8.67	76.25 ± 6.41	81.88 ± 2.77	49.70 ± 3.87	87.37 ± 4.23	71.01 ± 9.19	90.80 ± 6.27	88.73 ± 2.48	66.74 ± 6.17
PM	67.75 ± 5.14	75.99 ± 7.19	66.02 ± 6.83	78.62 ± 3.70	45.23 ± 4.21	66.07 ± 3.65	75.18 ± 8.38	64.15 ± 5.13	76.93 ± 3.56	43.47 ± 3.38
LR-L1	71.32 ± 7.47	66.84 ± 11.58	72.26 ± 11.03	78.41 ± 3.63	45.28 ± 4.44	91.69 ± 1.23	98.92 ± 0.63	91.85 ± 4.59	80.84 ± 3.05	**70.30 ± 5.36**
LR-L2	66.00 ± 3.00	69.39 ± 7.15	65.29 ± 3.61	75.16 ± 3.43	41.46 ± 3.62	81.08 ± 3.72	80.27 ± 5.97	81.25 ± 4.87	88.68 ± 2.46	59.84 ± 4.83
RF	80.97 ± 4.32	73.55 ± 10.49	82.53 ± 6.65	85.73 ± 2.97	57.63 ± 5.06	84.52 ± 3.75	69.76 ± 9.62	87.62 ± 5.98	87.73 ± 2.57	61.35 ± 4.71
Adaboost	73.41 ± 4.42	82.02 ± 6.17	71.60 ± 5.99	86.08 ± 2.45	51.94 ± 3.70	75.23 ± 9.06	80.91 ± 10.38	74.03 ± 12.72	87.78 ± 3.10	54.56 ± 7.16
GBT	83.14 ± 4.17	73.41 ± 7.95	85.18 ± 6.03	**88.07 ± 2.71**	**60.63 ± 5.32**	87.39 ± 2.93	71.35 ± 7.55	90.76 ± 3.97	**88.78 ± 3.29**	66.49 ± 5.64

Table 9. Comparing the performance of DA and various machine learning techniques and the dimension reduction stage for OFX and F1-F3.

	F1					F2					F3				
Method	Accuracy	Sensitivity	Specificity	AUC	F1-Score	Accuracy	Sensitivity	Specificity	AUC	F1-Score	Accuracy	Sensitivity	Specificity	AUC	F1-Score
DA	94.21 ± 0.66	65.07 ± 3.92	99.31 ± 0.28	82.19 ± 1.98	76.95 ± 3.04	94.21 ± 0.66	65.07 ± 3.92	99.31 ± 0.28	82.19 ± 1.98	76.95 ± 3.04	94.21 ± 0.66	65.07 ± 3.92	99.31 ± 0.28	82.19 ± 1.98	76.95 ± 3.04
SVM-RBF	84.38 ± 3.47	81.83 ± 5.43	84.82 ± 4.74	90.64 ± 1.83	61.41 ± 4.63	78.31 ± 11.72	71.73 ± 14.69	79.46 ± 19.73	71.74 ± 13.01	66.52 ± 10.56	92.89 ± 11.19	71.92 ± 6.49	96.55 ± 13.83	85.31 ± 10.26	78.27 ± 8.42
SVM-Linear	86.89 ± 3.52	79.34 ± 6.10	88.20 ± 4.83	90.52 ± 2.19	64.92 ± 5.35	94.21 ± 0.73	65.07 ± 3.92	99.31 ± 0.37	82.18 ± 2.01	76.95 ± 3.23	94.55 ± 0.77	72.64 ± 4.42	98.39 ± 0.81	88.14 ± 1.90	79.86 ± 3.00
PM	73.61 ± 3.71	85.80 ± 3.09	71.48 ± 4.49	86.97 ± 2.12	49.43 ± 3.53	91.01 ± 15.55	66.29 ± 8.00	95.34 ± 19.46	82.20 ± 2.05	74.76 ± 1.04	94.26 ± 1.21	70.85 ± 4.35	98.35 ± 1.34	87.04 ± 2.26	78.66 ± 3.94
LR-L1	95.03 ± 0.67	71.70 ± 6.70	90.74 ± 5.33	92.10 ± 1.71	69.12 ± 6.65	94.21 ± 0.77	65.02 ± 4.86	99.31 ± 0.34	82.21 ± 2.43	76.88 ± 3.64	95.03 ± 0.67	71.70 ± 3.89	99.11 ± 0.41	87.92 ± 1.60	**81.06 ± 2.83**
LR-L2	86.48 ± 4.56	79.04 ± 7.74	87.78 ± 6.43	91.81 ± 1.63	64.52 ± 6.57	94.21 ± 0.88	65.06 ± 5.01	99.31 ± 0.36	82.22 ± 2.57	76.90 ± 4.06	94.79 ± 0.71	72.18 ± 3.68	98.75 ± 0.53	88.50 ± 1.59	80.47 ± 2.78
RF	81.31 ± 4.07	81.54 ± 7.67	81.27 ± 5.82	90.00 ± 1.65	56.99 ± 4.28	94.21 ± 0.70	65.07 ± 4.76	99.31 ± 0.32	**82.22 ± 2.33**	**76.91 ± 3.32**	94.61 ± 0.78	70.96 ± 4.57	98.75 ± 0.58	**88.58 ± 1.86**	79.62 ± 3.19
Adaboost	65.08 ± 13.03	91.40 ± 5.72	60.48 ± 16.04	90.11 ± 1.90	45.55 ± 8.34	87.76 ± 21.49	67.12 ± 10.69	91.36 ± 26.94	82.00 ± 2.45	72.29 ± 14.10	87.76 ± 21.49	67.12 ± 10.07	99.35 ± 0.34	82.00 ± 2.45	72.29 ± 14.09
GBT	89.12 ± 4.62	79.06 ± 6.94	90.88 ± 6.38	**92.33 ± 1.49**	**69.63 ± 7.20**	94.21 ± 0.68	65.06 ± 4.28	99.31 ± 0.29	82.18 ± 2.33	76.91 ± 3.40	94.81 ± 0.71	70.20 ± 4.35	99.11 ± 0.41	88.12 ± 2.00	80.04 ± 3.08

	SNMF					SPCA				
	SNMF-F1					SPCA-F1				
Method	Accuracy	Sensitivity	Specificity	AUC	F1-Score	Accuracy	Sensitivity	Specificity	AUC	F1-Score
SVM-RBF	66.87 ± 5.77	66.92 ± 6.92	66.86 ± 7.18	76.58 ± 3.80	37.95 ± 4.52	85.84 ± 18.26	73.63 ± 11.38	87.98 ± 22.81	87.97 ± 14.01	67.09 ± 12.38
SVM-Linear	72.04 ± 3.57	68.22 ± 5.27	72.70 ± 4.55	79.65 ± 2.08	42.24 ± 3.11	92.05 ± 2.46	70.96 ± 6.78	95.74 ± 3.51	91.21 ± 1.90	73.08 ± 5.22
PM	70.14 ± 2.02	83.81 ± 4.23	67.75 ± 2.46	82.72 ± 2.09	45.56 ± 2.19	58.83 ± 4.02	82.65 ± 4.78	54.66 ± 5.01	78.93 ± 2.88	37.55 ± 2.79
LR-L1	62.54 ± 3.53	65.24 ± 7.13	62.07 ± 4.82	68.94 ± 3.01	34.16 ± 2.45	94.23 ± 0.81	65.38 ± 4.30	99.28 ± 5.48	87.43 ± 3.52	**77.08 ± 3.42**
LR-L2	62.66 ± 2.81	68.41 ± 4.44	61.66 ± 3.55	72.25 ± 2.34	35.34 ± 2.02	86.94 ± 1.79	79.04 ± 4.23	88.33 ± 2.28	90.84 ± 1.54	64.44 ± 3.31
RF	84.40 ± 3.56	75.82 ± 9.08	85.89 ± 5.32	90.50 ± 1.76	59.55 ± 4.36	86.83 ± 3.05	75.22 ± 7.67	88.86 ± 4.54	90.47 ± 2.02	63.38 ± 4.45
Adaboost	78.24 ± 3.72	84.30 ± 4.60	77.18 ± 4.73	90.16 ± 1.91	53.89 ± 4.12	82.27 ± 10.25	79.91 ± 10.15	82.68 ± 13.63	90.93 ± 2.06	60.63 ± 11.12
GBT	87.09 ± 3.42	75.45 ± 6.17	89.13 ± 4.70	**91.15 ± 1.61**	**64.13 ± 5.77**	91.06 ± 2.64	72.70 ± 6.16	94.27 ± 3.78	**91.61 ± 1.82**	71.25 ± 5.06

Table 10. Comparing the performance of DA and various machine learning techniques and the dimension reduction stage for KAN and F1-F3.

	F1					F2					F3				
Method	Accuracy	Sensitivity	Specificity	AUC	F1-Score	Accuracy	Sensitivity	Specificity	AUC	F1-Score	Accuracy	Sensitivity	Specificity	AUC	F1-Score
DA	94.79 ± 0.77	72.31 ± 5.40	97.61 ± 0.65	84.96 ± 2.68	75.54 ± 3.82	94.79 ± 0.77	72.31 ± 5.40	97.61 ± 0.65	84.96 ± 2.68	75.54 ± 3.82	94.79 ± 0.77	72.31 ± 5.40	97.61 ± 0.65	84.96 ± 2.68	75.54 ± 3.82
SVM-RBF	91.50 ± 3.35	80.11 ± 5.94	92.92 ± 4.19	91.50 ± 2.44	68.96 ± 8.27	52.98 ± 11.82	86.10 ± 14.39	48.81 ± 14.67	53.33 ± 34.53	47.83 ± 17.94	94.61 ± 2.09	77.16 ± 6.27	96.81 ± 2.37	89.82 ± 3.02	76.61 ± 6.53
SVM-Linear	90.50 ± 3.82	82.15 ± 6.13	91.55 ± 4.64	91.85 ± 2.38	67.08 ± 8.31	94.79 ± 0.77	72.32 ± 4.96	97.61 ± 0.63	81.16± 2.58	75.57 ± 3.71	95.57 ± 1.28	78.77 ± 4.46	97.69 ± 1.55	90.16 ± 2.88	80.11 ± 4.63
PM	84.76 ± 2.84	82.83 ± 6.05	85.00 ± 3.32	89.38 ± 3.41	55.15 ± 5.07	94.78 ± 0.99	71.53 ± 7.64	97.70 ± 0.85	85.64 ± 3.09	75.21 ± 5.48	89.21 ± 14.19	77.87 ± 8.30	90.63 ± 16.47	87.94 ± 3.91	68.52 ± 14.71
LR-L1	94.43 ± 2.18	77.89 ± 6.37	96.52 ± 2.81	91.96 ± 2.67	76.41 ± 6.45	94.76 ± 0.85	71.95 ± 5.28	97.63 ± 0.72	85.53 ± 2.71	75.37 ± 4.02	96.38 ± 1.96	77.26 ± 6.22	98.78 ± 2.17	90.01 ± 3.17	82.98 ± 6.01
LR-L2	92.05 ± 4.10	80.41 ± 6.48	93.48± 4.93	**92.49 ± 2.93**	70.86 ± 9.95	94.77± 0.76	71.94 ± 5.28	97.64 ± 0.71	85.62 ± 2.15	75.44 ± 3.50	94.22 ± 2.54	77.30 ± 6.69	96.34 ± 3.20	**90.87 ± 2.96**	75.73 ± 7.24
RF	88.82 ± 5.10	80.41 ± 7.20	89.88 ± 6.19	91.32 ± 2.54	63.28 ± 8.78	95.38 ± 1.03	64.63 ± 7.53	99.24 ± 0.80	85.57 ± 3.28	75.58 ± 5.88	96.05 ± 1.28	76.37 ± 5.11	98.53 ± 1.31	90.64 ± 2.63	81.36 ± 5.17
Adaboost	69.99 ± 15.91	87.11 ± 5.70	67.84 ± 6.05	90.44 ± 2.31	41.57 ± 7.15	95.40 ± 0.88	65.70 ± 6.54	99.13 ± 0.80	85.65 ± 2.89	76.04 ± 4.84	92.50 ± 0.86	73.01 ± 6.36	99.45 ± 0.84	88.78 ± 2.32	82.25 ± 4.40
GBT	94.95 ± 2.03	78.27 ± 6.80	97.04 ± 2.43	91.72 ± 2.88	**78.03 ± 6.53**	95.38 ± 0.78	65.36 ± 5.42	99.15 ± 0.76	**85.65 ± 2.62**	**75.89 ± 4.02**	96.65 ± 0.84	74.78 ± 6.62	99.40 ± 0.69	90.02 ± 2.86	**83.20 ± 4.45**

	SNMF					SPCA				
	SNMF-F1					SPCA-F1				
Method	Accuracy	Sensitivity	Specificity	AUC	F1-Score	Accuracy	Sensitivity	Specificity	AUC	F1-Score
SVM-RBF	83.66 ± 4.68	73.51 ± 8.18	84.94 ± 5.47	86.76 ± 4.03	50.97 ± 7.00	91.05 ± 11.73	79.10 ± 6.81	92.55 ± 13.65	90.51 ± 6.28	70.35 ± 9.72
SVM-Linear	84.48 ± 3.45	75.52 ± 8.47	85.61 ± 4.66	88.04 ± 2.45	52.61 ± 4.67	93.53 ± 2.28	78.76 ± 6.71	95.39 ± 3.03	91.68 ± 2.50	73.76 ± 6.07
PM	79.59 ± 3.84	78.19 ± 6.56	79.77 ± 4.75	86.34 ± 2.91	46.55 ± 4.89	86.14 ± 3.61	75.78 ± 8.26	87.44 ± 4.40	85.63 ± 3.92	55.57 ± 6.44
LR-L1	75.13 ± 4.24	68.98 ± 8.90	75.90 ± 5.41	78.53 ± 3.41	38.51 ± 4.19	95.56 ± 0.71	62.72 ± 5.92	99.68 ± 0.26	85.07 ± 3.66	**75.77 ± 4.65**
LR-L2	71.57 ± 5.47	74.48 ± 7.48	71.21 ± 6.25	81.17 ± 4.65	37.37 ± 5.18	93.14 ± 1.40	79.89 ± 5.03	94.81 ± 1.58	90.52 ± 2.53	72.36 ± 4.48
RF	91.33 ± 4.05	79.35 ± 6.50	92.84 ± 4.91	**91.87 ± 2.43**	**68.63 ± 9.24**	92.85 ± 3.25	79.68 ± 7.55	92.84 ± 4.91	**92.08 ± 2.42**	72.46 ± 7.84
Adaboost	81.74 ± 3.31	85.04 ± 5.28	81.32 ± 3.92	90.43 ± 2.74	51.35 ± 4.67	85.91 ± 3.93	84.63 ± 4.91	81.32 ± 3.92	91.65 ± 2.89	57.98 ± 6.43
GBT	91.41 ± 3.60	77.59 ± 7.23	93.14 ± 4.58	91.01 ± 2.47	68.00 ± 7.61	93.22 ± 2.65	78.29 ± 6.28	93.14 ± 4.58	91.91 ± 2.04	72.82 ± 7.27

Table 11. Comparing the performance of DA and various machine learning techniques and the dimension reduction stage for CAP and F1-F3.

F1						F2					F3				
Method	Accuracy	Sensitivity	Specificity	AUC	F1-Score	Accuracy	Sensitivity	Specificity	AUC	F1-Score	Accuracy	Sensitivity	Specificity	AUC	F1-Score
DA	90.35 ± 1.00	59.68 ± 5.84	93.87 ± 0.88	76.78 ± 2.96	56.00 ± 4.51	90.35 ± 1.00	59.68 ± 5.84	93.87 ± 0.88	76.78 ± 2.96	56.00 ± 4.51	90.35 ± 1.00	59.68 ± 5.84	93.87 ± 0.88	76.78 ± 2.96	56.00 ± 4.51
SVM-RBF	84.68 ± 7.12	68.83 ± 9.28	86.50 ± 8.86	84.91 ± 3.01	50.83 ± 10.08	74.22 ± 1.92	67.30 ± 6.80	75.02 ± 6.67	69.95 ± 3.47	55.92 ± 5.17	74.74 ± 4.39	66.60 ± 8.92	75.67 ± 9.73	73.59 ± 5.79	53.81 ± 8.69
SVM-Linear	84.54 ± 6.35	66.83 ± 7.82	86.58 ± 7.57	82.68 ± 3.66	48.96 ± 8.56	94.52 ± 0.54	57.78 ± 4.69	98.74 ± 0.39	78.23 ± 2.39	**68.39 ± 3.68**	91.68 ± 3.35	60.70 ± 6.42	95.24 ± 3.94	79.40 ± 4.10	61.27 ± 8.71
PM	72.81 ± 4.36	79.02 ± 5.80	72.10 ± 5.24	83.80 ± 2.65	37.74 ± 3.11	83.23 ± 2.83	62.70 ± 5.59	85.59 ± 6.01	**78.51 ± 3.40**	60.96 ± 5.70	78.57 ± 6.97	69.81 ± 6.41	79.58 ± 5.26	83.58 ± 2.30	47.84 ± 8.61
LR-L1	89.62 ± 5.43	63.08 ± 7.95	92.67 ± 6.80	84.57 ± 3.01	**58.08 ± 9.69**	94.50 ± 0.67	57.40 ± 5.21	98.76 ± 0.33	78.03 ± 2.76	68.16 ± 4.51	92.29 ± 4.40	58.83 ± 7.07	96.13 ± 5.26	83.52 ± 2.62	62.73 ± 8.50
LR-L2	85.57 ± 7.55	67.52 ± 8.61	87.65 ± 9.23	**85.33 ± 2.56**	52.38 ± 11.08	94.52 ± 0.73	57.71 ± 5.51	98.75 ± 0.40	78.02 ± 2.92	68.36 ± 4.79	91.20 ± 6.41	60.48 ± 8.60	94.74 ± 7.74	**84.08 ± 2.99**	61.63 ± 10.53
RF	82.22 ± 7.42	69.17 ± 11.32	83.73 ± 9.37	84.15 ± 3.00	46.58 ± 7.82	94.41 ± 1.06	57.71 ± 5.34	98.63 ± 0.96	78.06 ± 2.79	68.05 ± 5.11	92.87 ± 2.44	57.49 ± 7.86	96.89 ± 3.01	83.76 ± 3.19	62.88 ± 7.55
Adaboost	53.79 ± 6.50	80.89 ± 7.52	50.68 ± 8.08	84.29 ± 2.22	37.33 ± 7.92	70.89 ± 1.78	69.11 ± 6.68	71.09 ± 3.27	78.15 ± 2.31	54.10 ± 5.37	78.95 ± 6.22	65.46 ± 7.28	80.50 ± 5.77	83.19 ± 3.20	52.01 ± 10.88
GBT	88.14 ± 5.93	63.78 ± 9.54	90.94 ± 7.47	84.72 ± 2.98	54.99 ± 9.30	94.52 ± 0.61	57.78 ± 5.34	98.74 ± 0.43	78.08 ± 2.65	68.39 ± 4.22	93.96 ± 1.01	57.84 ± 5.74	98.11 ± 1.04	83.79 ± 3.23	**66.40 ± 4.95**

SNMF						SPCA				
SNMF-F1						SPCA-F1				
Method	Accuracy	Sensitivity	Specificity	AUC	F1-Score	Accuracy	Sensitivity	Specificity	AUC	F1-Score
SVM-RBF	74.44 ± 4.86	66.83 ± 7.69	75.32 ± 5.49	78.85 ± 3.57	35.48 ± 5.23	49.07 ± 6.41	76.95 ± 7.24	45.86 ± 6.73	53.54 ± 4.89	35.33 ± 10.30
SVM-Linear	77.56 ± 3.80	69.59 ± 7.65	78.48 ± 4.62	81.38 ± 3.09	39.28 ± 3.95	81.47 ± 4.11	65.24 ± 5.53	83.33 ±6.05	81.10 ± 6.89	52.01 ± 7.71
PM	75.80 ± 5.13	72.22 ± 6.44	76.21 ± 6.23	81.47 ± 2.44	38.68 ± 4.49	65.14 ± 3.11	74.86 ± 7.28	64.02 ± 4.07	75.75 ± 2.52	30.69 ± 1.65
LR-L1	90.35 ± 5.16	57.24 ± 7.60	94.16 ± 6.17	82.59 ± 3.24	**57.47 ± 10.86**	94.47 ± 0.68	56.51 ± 5.91	98.83 ± 3.75	77.67 ± 2.95	**67.66 ± 4.84**
LR-L2	67.93 ± 3.98	62.29 ± 7.95	68.57 ± 4.90	73.71 ± 3.24	28.71 ± 2.98	89.82 ± 2.04	64.44 ± 6.02	92.74 ± 2.52	**85.46 ± 2.02**	56.90 ± 4.61
RF	87.51 ± 5.39	63.71 ± 8.54	90.24 ± 6.71	**84.40 ± 2.60**	53.25 ± 8.71	87.51 ± 6.01	63.75 ± 7.25	90.24 ± 7.12	84.34 ± 2.90	53.72 ± 10.37
Adaboost	76.53 ± 3.37	72.25 ± 5.96	77.02 ± 4.04	82.57 ± 3.31	39.05 ± 3.45	82.69 ± 8.61	68.86 ± 9.01	84.28 ± 10.46	84.28 ± 10.46	47.98 ± 9.91
GBT	88.22 ± 4.70	62.86 ± 7.32	91.37 ± 5.69	83.46 ± 2.72	54.05 ± 8.62	90.05 ± 3.62	60.98 ± 8.11	93.39 ± 4.51	84.36 ± 2.87	56.94 ± 7.83

Table 12. Comparing the performance of DA and various machine learning techniques and the dimension reduction stage for CIP and F1-F3.

	F1					F2					F3				
Method	Accuracy	Sensitivity	Specificity	AUC	F1-Score	Accuracy	Sensitivity	Specificity	AUC	F1-Score	Accuracy	Sensitivity	Specificity	AUC	F1-Score
DA	92.57 ± 1.61	46.65 ± 10.10	99.24 ± 0.89	72.95 ± 5.17	60.97 ± 10.15	92.57 ± 1.61	46.65 ± 10.10	99.24 ± 0.89	72.95 ± 5.17	60.97 ± 10.15	92.57 ± 1.61	46.65 ± 10.10	99.24 ± 0.89	72.95 ± 5.17	60.97 ± 10.15
SVM-RBF	85.53 ± 7.38	77.99 ± 13.99	86.62 ± 9.04	88.08 ± 6.69	59.84 ± 12.09	41.29 ± 38.38	79.43 ± 26.90	54.42 ± 22.95	54.52 ± 22.95	35.79 ± 19.01	68.53 ± 34.50	79.63 ± 16.39	66.84 ± 26.78	68.35 ± 33.71	53.20 ± 20.84
SVM-Linear	86.37 ± 7.39	77.07 ± 12.67	87.72 ± 8.85	88.17 ± 5.96	**61.13 ± 12.35**	89.28 ± 15.66	48.15 ± 14.05	95.26 ± 19.46	72.79 ± 4.61	58.77 ± 11.09	92.03 ± 3.31	73.34 ± 13.56	94.74 ± 3.86	87.10 ± 6.21	**70.70 ± 11.57**
PM	55.42 ± 29.61	79.66 ± 23.76	52.11 ± 23.76	86.96 ± 5.87	39.93 ± 17.87	36.16 ± 35.99	80.82 ± 29.63	29.79 ± 35.20	72.42 ± 5.76	30.93 ± 13.91	81.65 ± 22.01	75.24 ± 14.26	82.61 ± 26.69	86.76 ± 5.21	60.97 ± 17.01
LR-L1	83.25 ± 6.34	73.55 ± 12.99	84.65 ± 7.89	86.15 ± 5.96	53.99 ± 9.86	66.30 ± 36.98	58.50 ± 30.07	67.58 ± 37.58	71.53 ± 6.38	43.50 ± 18.01	90.03 ± 2.93	63.76 ± 13.52	93.85 ± 3.97	84.66 ± 5.11	61.99 ± 8.79
LR-L2	84.67 ± 6.02	79.86 ± 9.98	85.37 ± 7.65	**89.53 ± 4.06**	58.49 ± 9.30	56.93± 39.46	67.72 ± 29.52	55.56 ± 38.86	**73.06 ± 5.47**	41.58 ± 18.73	89.55 ± 8.99	73.35 ± 12.32	91.91 ± 10.67	87.37 ± 5.60	66.19 ± 10.75
RF	86.39 ± 7.31	67.79 ± 14.67	89.07 ± 9.55	87.20 ± 4.94	58.04 ± 11.16	89.17 ± 15.75	95.26 ± 19.46	86.92 ± 17.87	72.75 ± 5.94	57.78 ± 13.60	93.22 ± 2.59	61.98 ± 14.45	97.77 ± 2.68	**87.72 ± 5.41**	69.54 ± 11.03
Adaboost	32.41 ± 27.18	94.69 ± 8.33	23.37 ± 32.09	86.81 ± 5.98	29.62 ± 11.30	19.09 ± 21.59	95.87 ± 14.23	18.33 ± 19.26	73.01 ± 5.38	25.63 ± 10.69	86.67 ± 17.48	66.05 ± 17.09	89.64 ± 21.51	84.92 ± 5.31	62.67 ± 15.77
GBT	87.56 ± 5.81	70.49 ± 13.34	90.02 ± 6.83	87.35 ± 5.42	60.35 ± 12.15	92.35 ± 1.57	44.98 ± 9.58	99.24 ± 0.94	72.43 ± 4.83	**59.44 ± 9.70**	93.29 ± 2.37	64.46 ± 15.35	97.49 ± 2.33	87.62 ± 4.44	70.28 ± 12.30

	SNMF					SPCA				
	SNMF-F1					SPCA-F1				
Method	Accuracy	Sensitivity	Specificity	AUC	F1-Score	Accuracy	Sensitivity	Specificity	AUC	F1-Score
SVM-RBF	52.38 ± 26.98	70.69 ± 49.67	22.22 ± 10.64	62.64 ± 23.04	29.93 ± 8.80	59.43 ± 36.90	75.96 ± 23.84	57.01 ± 29.02	63.25 ± 31.45	44.34 ± 20.18
SVM-Linear	75.04 ± 8.13	64.90 ± 19.38	76.54 ± 11.18	80.30 ± 5.90	40.17 ± 7.37	79.39 ± 2.28	67.98 ± 16.75	87.08 ± 17.41	79.81 ± 19.71	52.60 ± 13.07
PM	69.85 ± 7.55	74.08 ± 11.26	69.17 ± 9.02	78.95 ± 5.12	39.33 ± 6.94	74.12 ± 11.05	65.57 ± 16.78	75.37 ± 14.57	80.40 ± 5.35	41.31 ± 9.61
LR-L1	46.12 ± 26.99	59.55 ± 44.269	16.19 ± 10.90	52.91 ± 7.69	25.37 ± 8.91	80.32 ± 27.44	43.98 ± 25.16	85.70 ± 29.34	67.60 ± 5.98	45.86 ± 15.52
LR-L2	57.97 ± 14.44	64.34 ± 57.10	19.23 ± 14.20	67.78 ± 7.80	26.45 ± 8.76	87.70 ± 3.99	58.03 ± 17.83	92.04 ± 6.37	84.87 ± 6.37	54.13 ± 10.32
RF	83.93 ± 5.74	62.33 ± 15.44	87.09 ± 7.69	82.63 ± 6.16	49.91 ± 9.93	84.17 ± 6.01	68.37 ± 14.37	86.46 ± 7.80	85.71 ± 5.66	53.23 ± 10.03
Adaboost	77.61 ± 6.37	74.48 ± 13.96	78.06 ± 7.84	**84.51 ± 5.69**	46.37 ± 8.66	76.30 ± 6.94	75.97 ± 16.25	76.33 ± 9.00	85.52 ± 6.06	45.28 ± 10.74
GBT	81.86 ± 7.25	68.75 ± 11.74	83.78 ± 8.52	83.47 ± 5.99	**50.58 ± 10.80**	87.50 ± 4.39	71.38 ± 13.07	89.81 ± 5.84	**86.52 ± 6.74**	**59.86 ± 8.92**

supplementary C

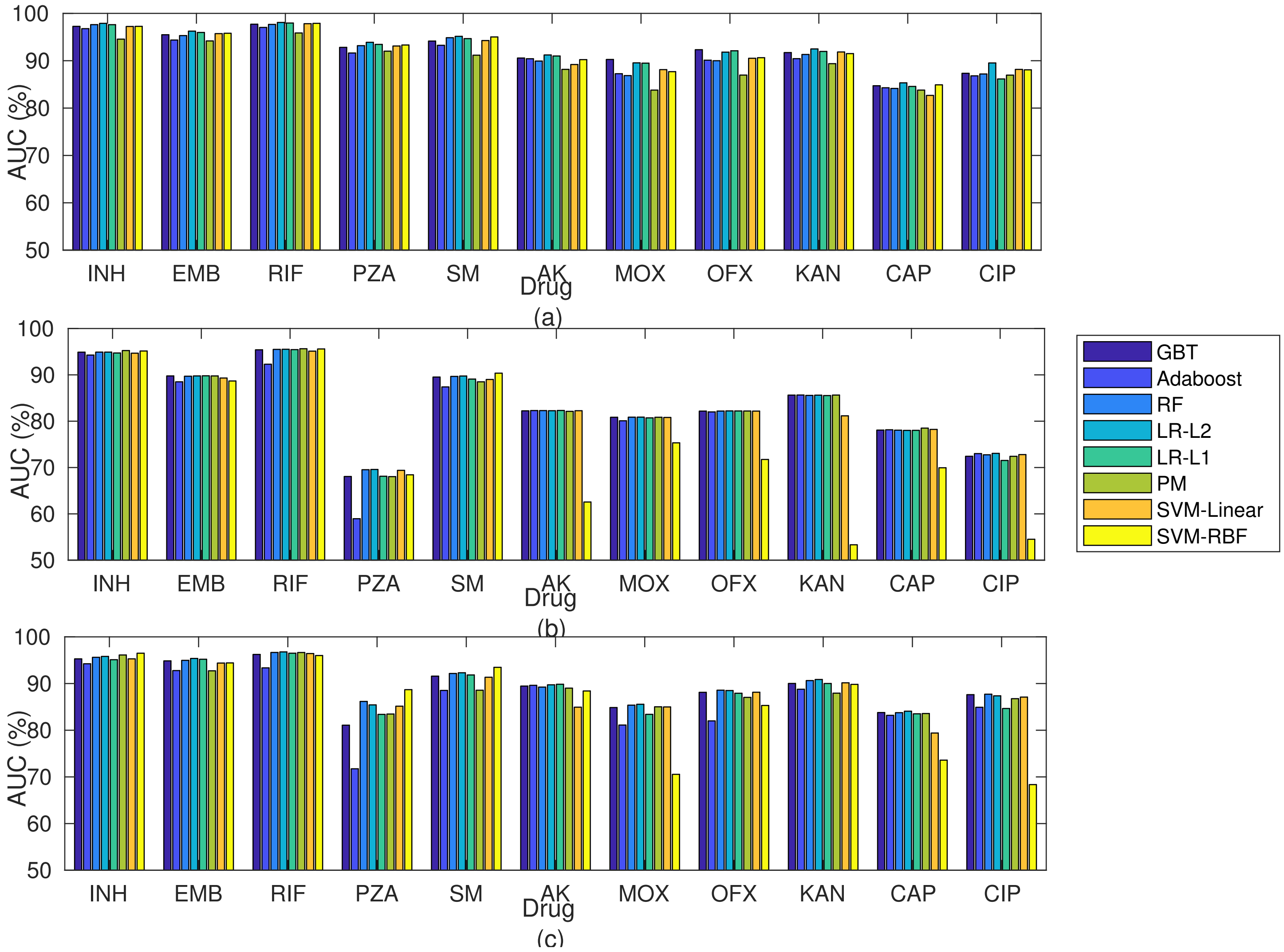

Fig. 1: Classification performance (AUC%) considering six machine learning classifiers across 11 anti-TB drugs and three feature spaces (a) F1, (b) F2, and (c) F3.

F1-Score: Considering F1 led to the best performing model for INH and PZA, F2 for AK and CAP and F3 for other drugs. Most models had similar F1-score based on F1 with exceptions of (F1 + PM) and (F1 + Adaboost) that resulted in good F1-Score only for INH, EMB, RIF, and PZA with addition to SM for Adaboost. Similarly, (F2 + Adaboost) performed similar to other techniques only for INH, EMB, RIF, AK, and KAN while (F2 + SVM-RBF) had lower F1-Score for AK, OFX, and KAN and (F1 + PM) for CAP and CIP. Most techniques performed the same considering F3. For all drugs, much higher F1-Score have obtained considering the top performed classifiers (supplementary C). The F1-Score improved by up to 12% for CAP (F2 + SVM-Linear), 9% for PZA and CIP (F1/F3 + SVM-Linear), 7% for KAN (F3 + GBT), 4% for OFX and up to 2% for other drugs compared to DA. The F1-Score is specially increased for PZA, KAN, and CAP that less then 10% isolates are resistant.

Most techniques performed similarly considering SPCA/SNMF except for considering (F1 + SPCA + PM) for all drugs, (F1 + SPCA + SVM-RBF) for AK and CAP, and (F1 + SPCA + Adaboost) for MOX, OFX, KAN, CAP, and CIP in comparison with other models. Furthermore, (F1 + SNMF + GBT) and (F1 + SNMF + LR-L1) was the top performing models considering F1-Score. Moreover, adding SPCA for the dimension reduction step enhanced the F1-score by up to 12.54%, 4.61%, 7.45%, and 9.58% for AK, MOX, OFX, and CAP respectively compared to considering the whole F1.

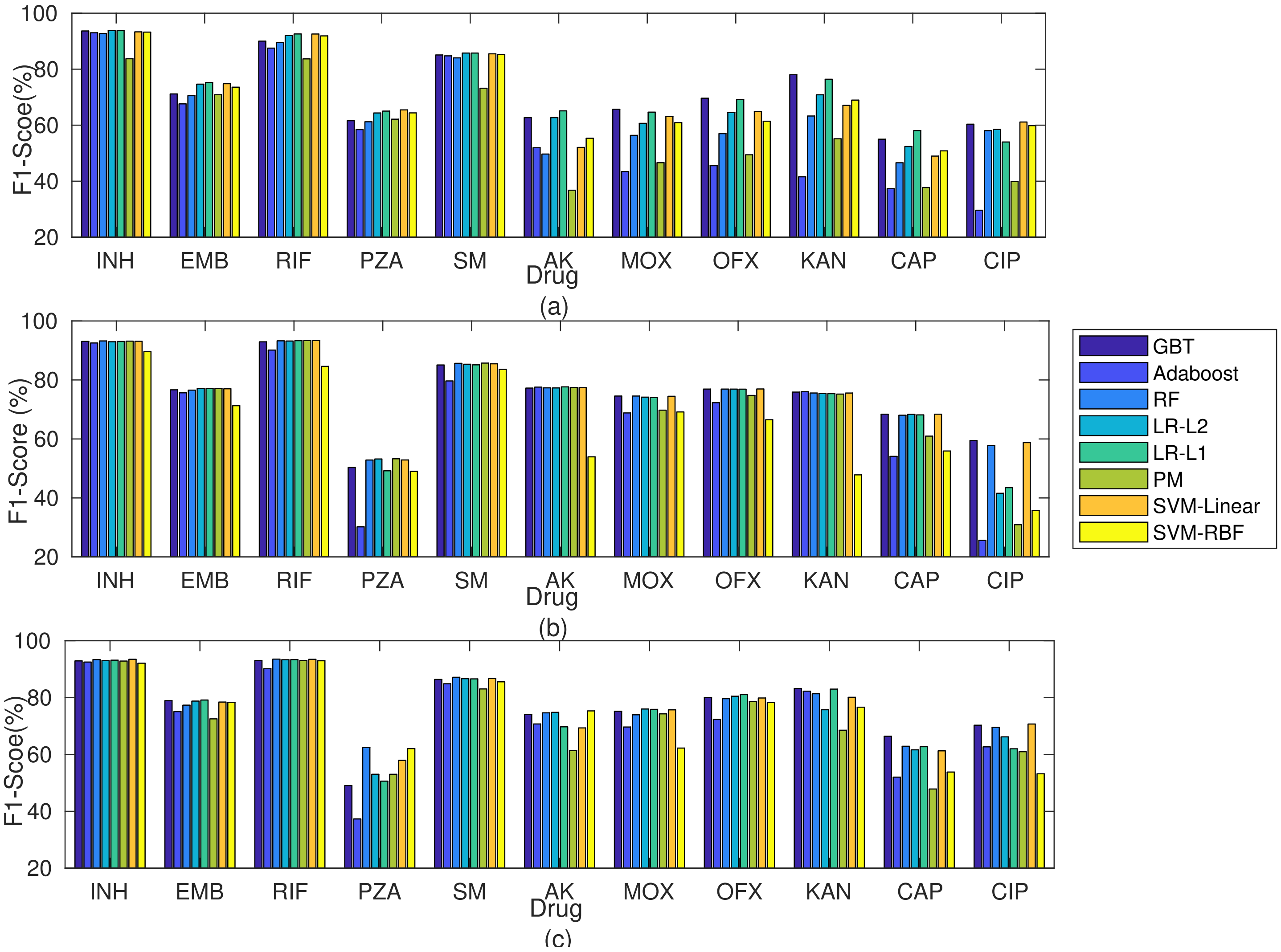

Fig. 2: Classification performance (F1-Score%) considering six machine learning classifiers across 11 anti-TB drugs and three feature spaces (a) F1, (b) F2, and (c) F3.

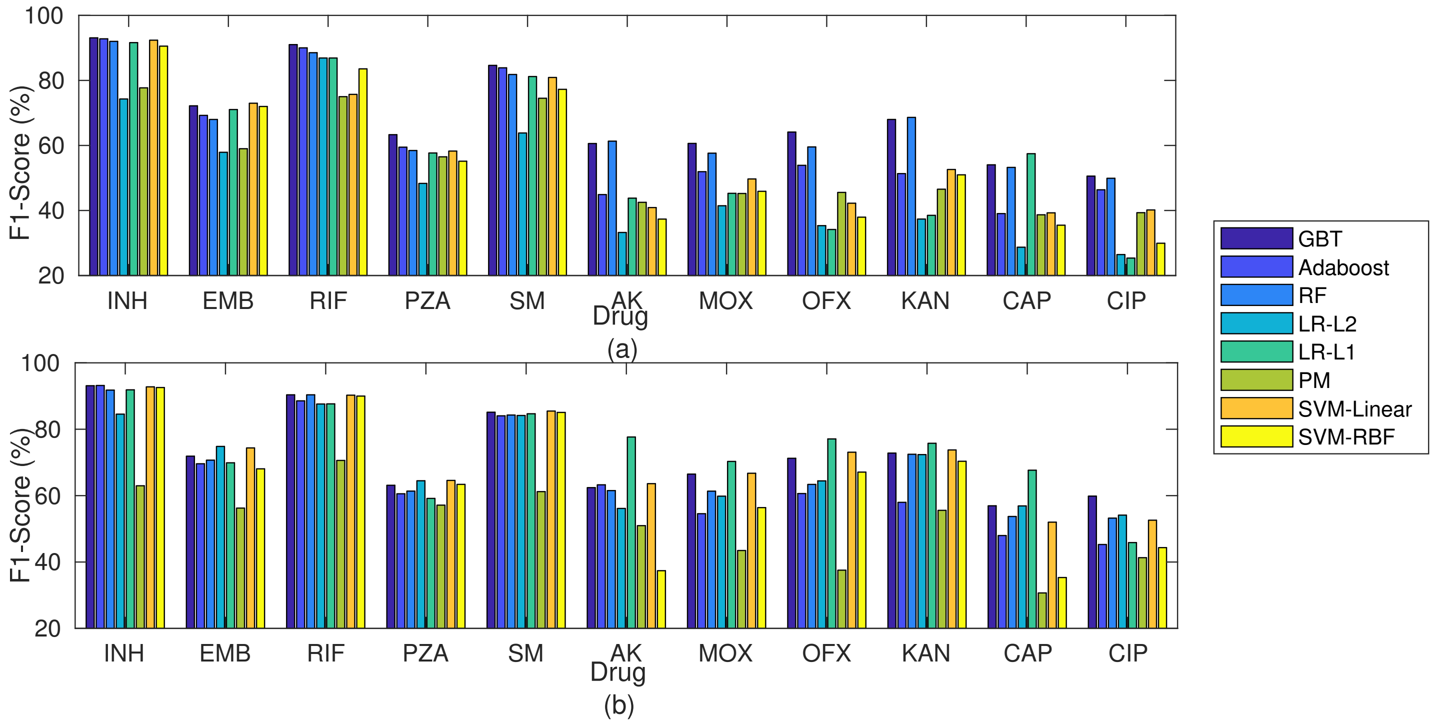

Fig. 3: Classification performance (F1-Score%) considering six machine learning classifiers across 11 anti-TB drugs and (a) SNMF-F1 and (b) SPCA-F1.

supplementary D

Table 13. Top 10 mutations ranked by LR-L1, LR-L2, and GBT; resistance/susceptible-associated mutations to each given drug are indicated in the boldface. The other mutations are either known to be related to other drugs or not in the library.

INH	EMB	RIF	PZA	SM	AK	OFX	MOX	KAN	CAP	CIP
LR-L1										
rrs_A1401G	**embB_M306V**	**rpoB_S450L**	pncA_L120P	**rpsL_K43R**	**rrs_A1401G**	gyrA_E21Q	gyrA_D94A	**rrs_A1401G**	**rrs_A1401G**	gyrA_G668D
**katG_S315T**	**embB_D328Y**	**rpoB_D435V**	rpsA_A381V	katG_S315T	gidB_E92D	gyrA_D94A	**gyrA_D94G**	**eis_C-12T**	pncA_C14G	gyrA_S95T
**fabG1_G-17T**	**embB_G406A**	**rpoB_S450W**	**pncA_Q10P**	**rpsL_K88R**	gyrA_D94A	**gyrA_D94G**	**gyrA_A90V**	**eis_G-37T**	**rrs_C1402T**	gyrA_E21Q
eis_C-12T	**embB_D1024N**	**rpoB_H445C**	pncA_G97S	**rrs_A514C**	rmlD_L282L	**gyrA_A90V**	**gyrA_D94N**	embA_Q38Q	iniC_T89I	gyrA_D94A
**katG_S315N**	**embB_Q497R**	**rpoB_H445Y**	**pncA_C138R**	pncA_Y103H	eis_C-14T	**gyrA_D94N**	**gyrA_D94Y**	**eis_C-14T**	pncA_A102P	**gyrA_D94G**
**fabG1_C-15T**	embB_Y319S	**rpoB_H445L**	**pncA_K96T**	inhA_I194T	embA_C-8T	**gyrA_D94Y**	**gyrA_S91P**	**eis_G-10A**	ndh_A209V	pncA_D136G
**fabG1_L203L**	**embB_D328G**	**rpoB_V170F**	pncA_H51D	**gidB_P75R**	gyrA_D94N	**gyrA_S91P**	**gyrA_G88C**	inhA_I21T	pncA_M175V	**gyrA_A90V**
rpoB_V170F	rrs_C513T	**rpoB_H445D**	**pncA_G97D**	gyrA_K542K	pncA_Q141P	**gyrA_G88C**	gyrB_E540D	rpoB_D435G	**tlyA_N236K**	gyrA_D94Y
**ahpC_C-54T**	**embA_C-11A**	pncA_H51D	**pncA_H57D**	**gidB_G69D**	embB_Y334H	gyrB_E540D	gyrB_D500N	embB_Y334H	gidB_G62G	**gyrA_S91P**
gidB_G71*	**embA_C-16G**	**rpoB_S450F**	**pncA_H57R**	rpoB_T52P	gidB_G62G	**gyrA_D94H**	**gyrA_D94H**	gyrB_G77S	embB_R507R	iniA_S501W
LR-L2										
**katG_S315T**	embB_Y319S	**rpoB_S450L**	katG_S315T	katG_S315T	**rrs_A1401G**	**gyrA_D94G**	**gyrA_D94G**	**rrs_A1401G**	**rrs_A1401G**	**gyrA_D94G**
**fabG1_C-15T**	**embB_M306V**	pncA_H51D	**pncA_H57D**	**rpsL_K43R**	gyrA_E21Q	**gyrA_A90V**	**gyrA_A90V**	**eis_G-10A**	**rrs_C1402T**	gyrA_D94A
**fabG1_L203L**	**embB_D328G**	**rpoB_H445Y**	pncA_L120P	**rpsL_K88R**	gidB_S100F	**gyrA_S91P**	**gyrA_D94Y**	**eis_C-14T**	embB_R507R	pncA_D136G
**katG_S315N**	**embB_M306I**	**rpoB_H445D**	katG_S315N	**rrs_A514C**	katG_S315T	**gyrA_D94A**	**gyrA_D94A**	**eis_G-37T**	gidB_G62G	**gyrA_A90V**
rpoB_S450L	**embB_Q497R**	**rpoB_D435V**	rpsA_A381V	rrs_C517T	eis_C-14T	**gyrA_G88C**	**gyrA_S91P**	gyrB_G77S	pncA_D12N	gyrA_S95T
rpoB_V170F	**embA_C-16G**	**rpoB_V170F**	pncA_H51D	inhA_I194T	pncA_T142A	**gyrA_D94N**	**gyrA_D94N**	gyrA_E21Q	gyrA_E21Q	embB_M306I
**fabG1_G-17T**	**embB_G406A**	**rpoB_S450W**	**pncA_G97D**	**gidB_P75R**	rpoB_C-61T	**gyrA_D94H**	**gyrA_D94H**	embA_Q38Q	ndh_Y108C	**gyrA_S91P**
rpoB_D435V	**embA_C-11A**	**rpoB_H445L**	**rpsA_A440T**	rrs_G878A	embB_Y334H	**gyrA_D94Y**	**gyrA_G88C**	embB_Y334H	iniC_T89I	katG_C-85T
rpoB_S450W	**embB_M306L**	**rpoB_H445R**	**pncA_Q10P**	gyrA_K542K	pncA_P62S	gyrA_E21Q	gyrB_D500N	**eis_C-12T**	rpoB_C-61T	rpoB_V168A
rpsA_A381V	**embB_D1024N**	**rpoB_S450F**	katG_V473L	**rrs_A906G**	gidB_G62G	gyrB_E540D	inhA_I21V	pncA_H57R	pncA_F13L	embB_D869E
GBT										
**katG_S315T**	katG_S315T	**rpoB_S450L**	katG_S315T	katG_S315T	**rrs_A1401G**	**gyrA_D94G**	**gyrA_D94G**	**rrs_A1401G**	**rrs_A1401G**	**gyrA_D94G**
**fabG1_C-15T**	**embB_M306V**	katG_S315T	**pncA_H57D**	**rpsL_K43R**	pncA_T142A	**gyrA_A90V**	**gyrA_A90V**	**eis_G-10A**	**rrs_C1402T**	gyrA_D94A
**fabG1_L203L**	rpoB_S450L	**rpoB_H445Y**	rpoB_S450L	**rpsL_K88R**	pncA_T135S	**gyrA_S91P**	**gyrA_D94Y**	**eis_C-14T**	embB_R507R	pncA_D136G
**katG_S315N**	**embB_M306I**	**rpoB_H445D**	embB_M306V	**rrs_A514C**	rrs_G1016C	**gyrA_D94A**	**gyrA_D94A**	**eis_G-37T**	gidB_G62G	**gyrA_A90V**
rpoB_S450L	**embB_Q497R**	**rpoB_D435V**	**pncA_A-11G**	rrs_C517T	embC_L121L	**gyrA_G88C**	**gyrA_S91P**	gyrB_G77S	pncA_D12N	rpoB_N437D
rpoB_V170F	**embA_C-16G**	**rpoB_V170F**	embB_M306I	rpoB_S450L	tlyA_E186E	**gyrA_D94N**	**gyrA_D94N**	gidB_G62G	katG_V423I	eis_G-10A
katG_G699E	**embB_G406A**	**rpoB_S450W**	embB_G406A	pncA_A38A	iniA_I415V	**gyrA_D94H**	**gyrA_D94H**	pncA_T142A	iniA_P177L	gyrA_G88C
rpoB_D435V	**embA_C-12T**	**rpoB_H445L**	embB_Q497R	**gidB_G69D**	embC_A931T	**gyrA_D94Y**	**gyrA_G88C**	embB_R507R	iniA_L320L	gyrB_S313R
**ahpC_C-52T**	**embB_M306L**	**rpoB_H445R**	**pncA_Q10P**	embB_M306V	**rrs_G1484T**	embA_C-12T	gyrB_D500N	**eis_C-12T**	pncA_A134D	embR_A224A
**katG_Q461P**	**embB_D1024N**	**rpoB_L452P**	**rrs_A1401G**	**rrs_A906G**	rpsA_R212R	katG_S315T	katG_S315T	eis_L241L	**rrs_G1484T**	rpoB_T229T

Supplementary E

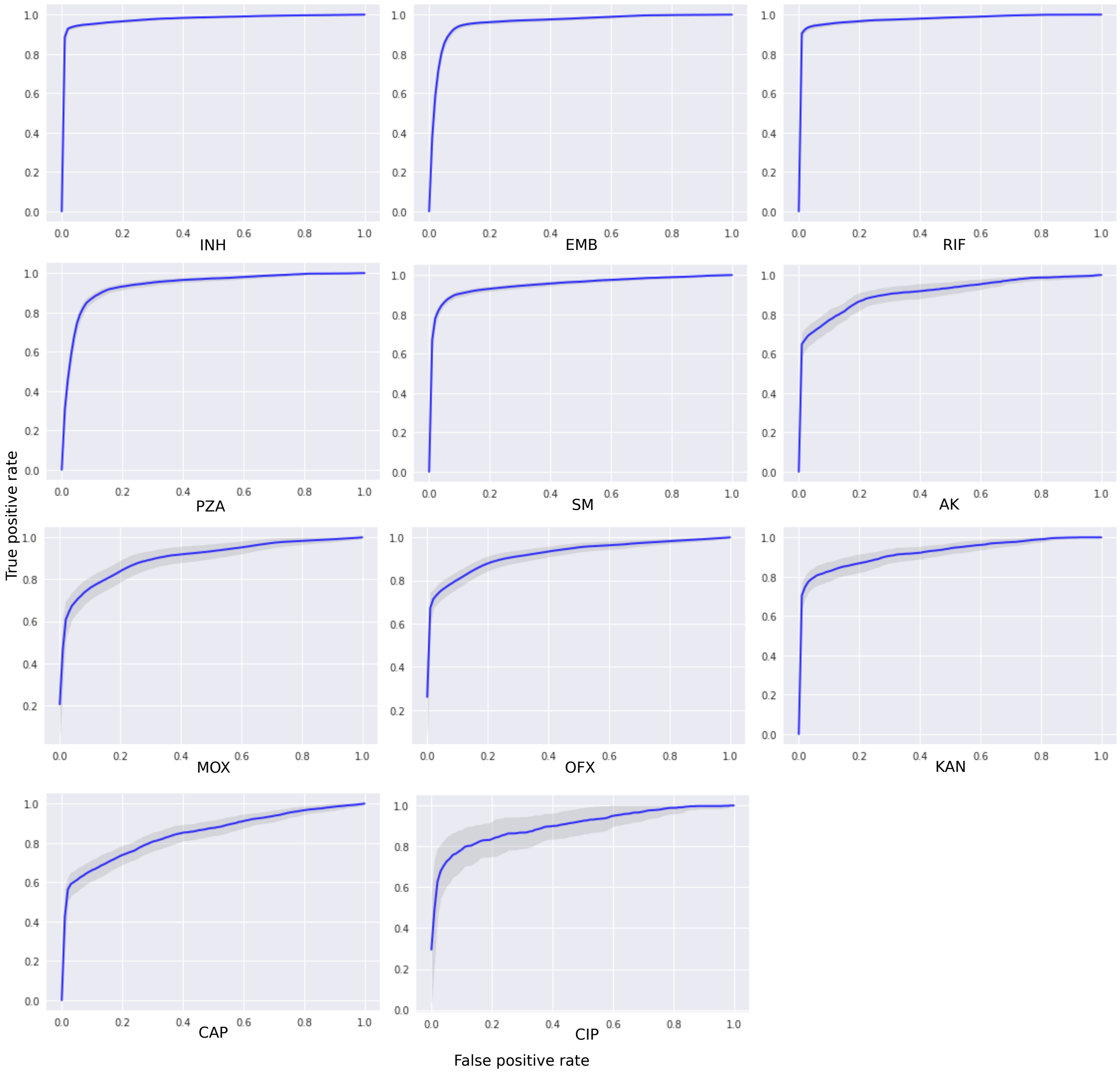

Fig. 4: Mean ROC (± STD) for the best performing model model for each drug on various test sets in various runs.

Supplementary F

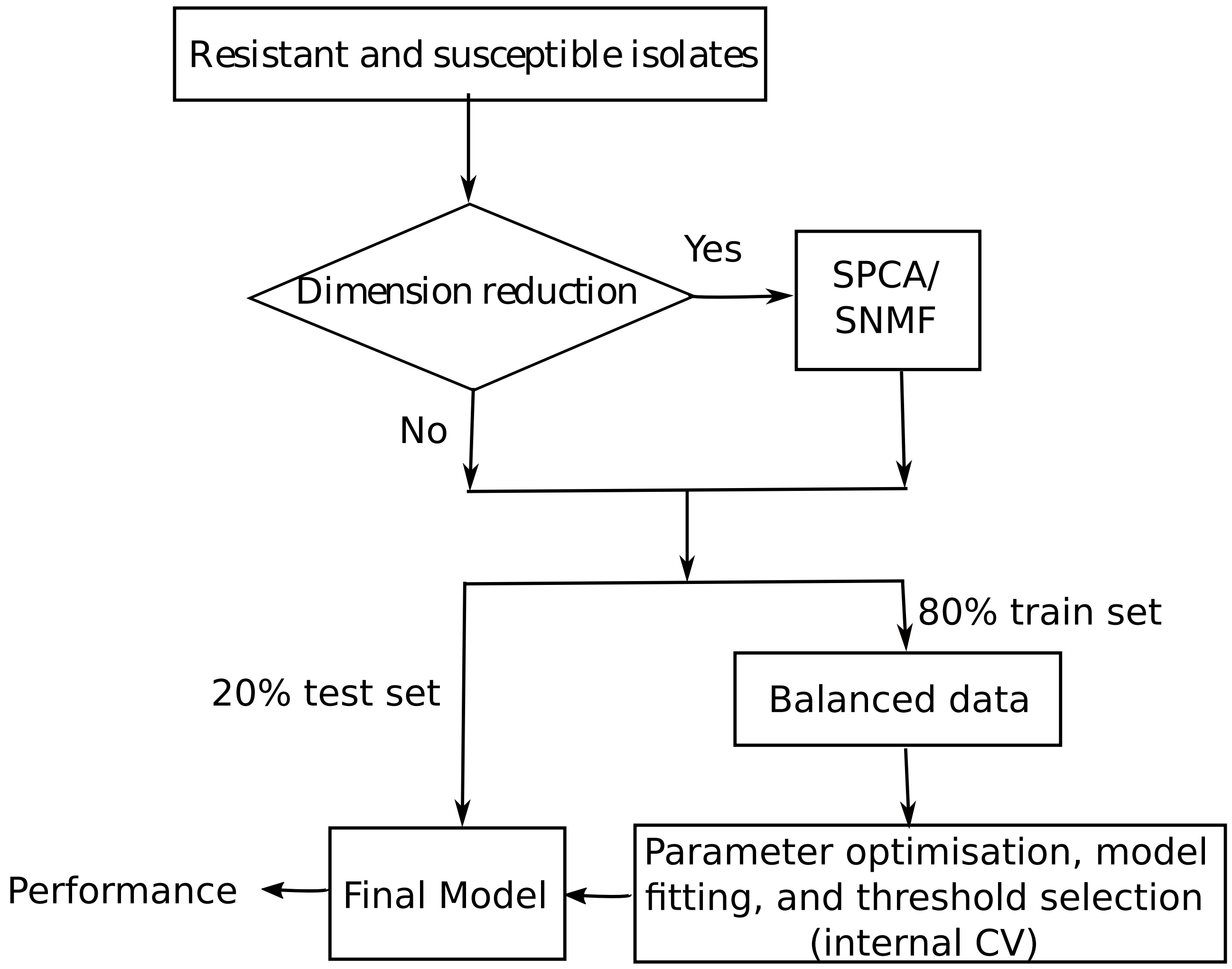

Fig. 5: The workflow for the examined classifiers.

Supplementary G

Table 14. A list of 23 candidate genes and their associated drugs.

Genes	Relevant drug
ahpC, fabG1, inhA, katG, ndh	INH
rpoB	RIF
embA, embB, embC, embR, iniA, iniC, manB, rmlD	EMB
pncA, rpsA	PZA
gyrA, gyrB	OFX, MOX, CIP
rpsL, gidB, rrs, tlyA	SM
gidB, rrs, tlyA	AK and CAP
gidB, rrs, tlyA, eis	KAN

Table 15. The number of features for each drug considering F3.

Drug	INH	EMB	RIF	PZA	SM	KAN	AK	CAP	CIP	OFX	MOX
Susceptible	678	2095	439	503	1075	1198	1029	1029	863	863	863

Supplementary H

This section compares the performance of (F1 + LR-L2/GBT) and (F1 + SNMF/SPCA + LR-L2/GBT). LR-L2 was the top performing classifier considering the whole feature space while GBT performed best after considering the dimension reduction stage. (F1 + SNMF/SPCA + GBT) in comparison with (F1 + GBT) improved AUC for INH, EMB, RIF, PZA, SM, KAN, and AK, sensitivity for INH, KAN, and CIP, and specificity for EMB, RIF, PZA, CAP, MOX, and OFX (p < 0.01). (F1 + SPCA + LR-L2) comparing with (F1 + LR-L2) resulted in higher AUC for CAP and AK, sensitivity for AK, MOX, and OFX, and specificity for INH, EMB, RIF, PZA, SM, KAN, CAP, CIP, and OFX (p < 0.01).

Table 16. Comparing GBT performance considering 11 drugs and F1/(F1 + SNMF/SPCA). Sensitivity, specificity, and AUC (mean ± standard error) are reported. Wilcoxon signed-rank test was used to calculate the p-value and $^\diamond$ indicates $p < 0.01$.

	GBT			SPCA/SNMF + Classifier			
Drugs	Sensitivity	Specificity	AUC	Dimension reduction method	Sensitivity	Specificity	AUC
INH	91.77 ± 1.03	98.41 ± 0.32	97.26 ± 0.41	SNMF	92.18$^\diamond$ ± 0.98	97.79 ± 0.42	97.65$^\diamond$ ± 0.35
EMB	92.68 ± 1.87	89.93 ± 1.35	95.49 ± 0.53	SNMF	92.15 ± 1.76	90.64$^\diamond$ ± 1.06	95.70$^\diamond$ ± 0.52
RIF	92.39 ± 1.34	96.27 ± 1.23	97.71 ± 0.35	SNMF	92.30 ± 1.43	96.69$^\diamond$ ± 0.77	97.79$^\diamond$ ± 0.39
PZA	88.49 ± 2.76	87.22 ± 1.86	92.82 ± 0.98	SNMF	88.14 ± 2.46	88.34$^\diamond$ ± 1.36	93.50$^\diamond$ ± 0.82
SM	87.90 ± 1.69	93.28 ± 1.21	94.15 ± 0.81	SNMF	87.45 ± 2.14	93.16 ± 1.40	95.09$^\diamond$ ± 0.61
AK	73.82 ± 7.72	92.68 ± 5.97	90.57 ± 2.67	SPCA	73.40 ± 8.07	92.60 ± 6.13	90.94$^\diamond$ ± 2.45
MOX	76.84 ± 9.29	87.19 ± 8.21	90.27 ± 2.96	SPCA	71.35 ± 7.55	90.76$^\diamond$ ± 3.97	88.78 ± 3.29
OFX	79.06 ± 6.94	90.88 ± 6.38	92.33 ± 1.49	SPCA	72.70 ± 6.16	94.27$^\diamond$ ± 3.78	91.61 ± 1.82
KAN	78.27 ± 6.80	97.04 ± 2.43	91.72 ± 2.88	SPCA	78.29$^\diamond$ ± 6.28	93.14 ± 4.58	91.91$^\diamond$ ± 2.04
CAP	63.78 ± 9.54	90.94 ± 7.47	84.72 ± 2.98	SPCA	60.98 ± 8.11	93.39$^\diamond$ ± 4.51	84.36 ± 2.87
CIP	70.49 ± 13.34	90.02 ± 6.83	87.35 ± 5.42	SPCA	71.38$^\diamond$ ± 13.07	89.81 ± 5.84	86.52 ± 6.74

Table 17. Comparing LR-L2 performance considering 11 drugs and F1/(F1 + SNMF/SPCA). Sensitivity, specificity, and AUC (mean ± standard error) are reported. Wilcoxon signed-rank test was used to calculate the p-value and $^\diamond$ indicates $p < 0.01$.

	LR-L2			SPCA/SNMF + Classifier			
Drugs	Sensitivity	Specificity	AUC	Dimension reduction method	Sensitivity	Specificity	AUC
INH	92.19 ± 0.94	98.38 ± 0.29	97.89 ± 0.38	SPCA	75.69 ± 1.39	98.74$^\diamond$ ± 0.22	96.52 ± 0.39
EMB	92.12 ± 1.84	91.89 ± 0.84	96.25 ± 0.54	SPCA	86.80 ± 1.67	93.30$^\diamond$ ± 0.55	95.57 ± 0.48
RIF	92.77 ± 1.28	97.45 ± 0.63	98.08 ± 0.32	SPCA	82.95 ± 1.54	98.15$^\diamond$ ± 0.27	97.10 ± 0.47
PZA	88.12 ± 2.65	88.91 ± 1.66	93.89 ± 0.80	SPCA	81.96 ± 2.35	90.70$^\diamond$ ± 0.56	92.30 ± 0.78
SM	87.40 ± 1.98	94.15 ± 1.23	95.15 ± 0.56	SPCA	81.37 ± 1.82	95.72$^\diamond$ ± 0.65	93.20 ± 0.78
AK	73.51 ± 9.09	92.20 ± 7.53	91.22 ± 2.35	SPCA	77.23$^\diamond$ ± 6.96	89.84 ± 3.05	91.37$^\diamond$ ± 2.36
MOX	80.08 ± 9.26	81.35 ± 9.29	89.55 ± 2.84	SPCA	80.27$^\diamond$ ± 5.97	81.25 ± 4.87	88.68 ± 2.46
OFX	79.04 ± 7.74	87.78 ± 6.43	91.81 ± 1.63	SPCA	79.04$^\diamond$ ± 4.23	88.33$^\diamond$ ± 2.28	90.84 ± 1.54
KAN	80.41 ± 6.48	93.48± 4.93	92.49 ± 2.93	SPCA	79.89 ± 5.03	94.81$^\diamond$ ± 1.58	90.52 ± 2.53
CAP	67.52 ± 8.61	87.65 ± 9.23	85.33 ± 2.56	SPCA	64.44 ± 6.02	92.74$^\diamond$ ± 2.52	85.46$^\diamond$ ± 2.02
CIP	79.86 ± 9.98	85.37 ± 7.65	89.53 ± 4.06	SPCA	58.03 ± 17.83	92.04$^\diamond$ ± 6.37	84.87 ± 6.37

Supplementary I

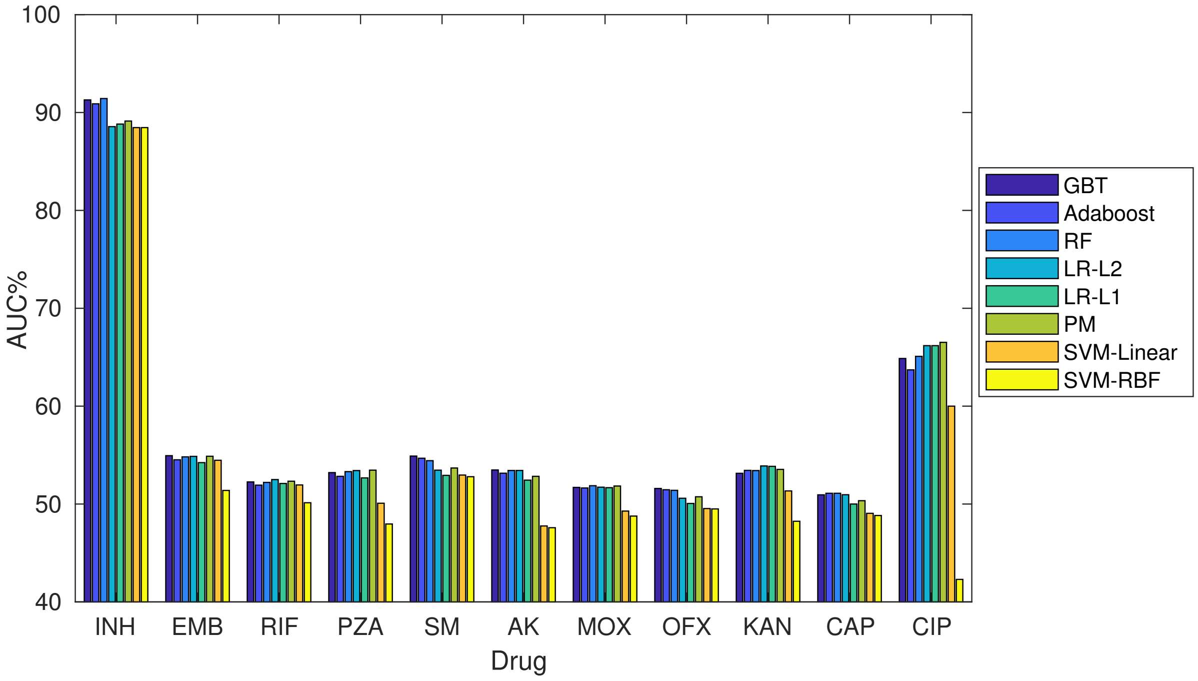

Fig. 6: Classification performance (AUC%) considering six machine learning classifiers across 11 anti-TB drugs and F1 + binary matrix factorisation (BMF).

Supplementary J

Here, we compared the performance of (F1 + SPCA/SNMF + GBT) for all drugs keeping 50, 100, and 150 components. Considering different number of components in (F1 + SNMF + GBT) resulted in similar performance for all drugs except for CIP and RIF. Keeping lower components showed to improve the performance of CIP and RIF. Considering higher number of components in (F1 + SPCA + GBT) indicated no effect on the performance for most of drugs but can improve the performance for MOX and OFX drugs.

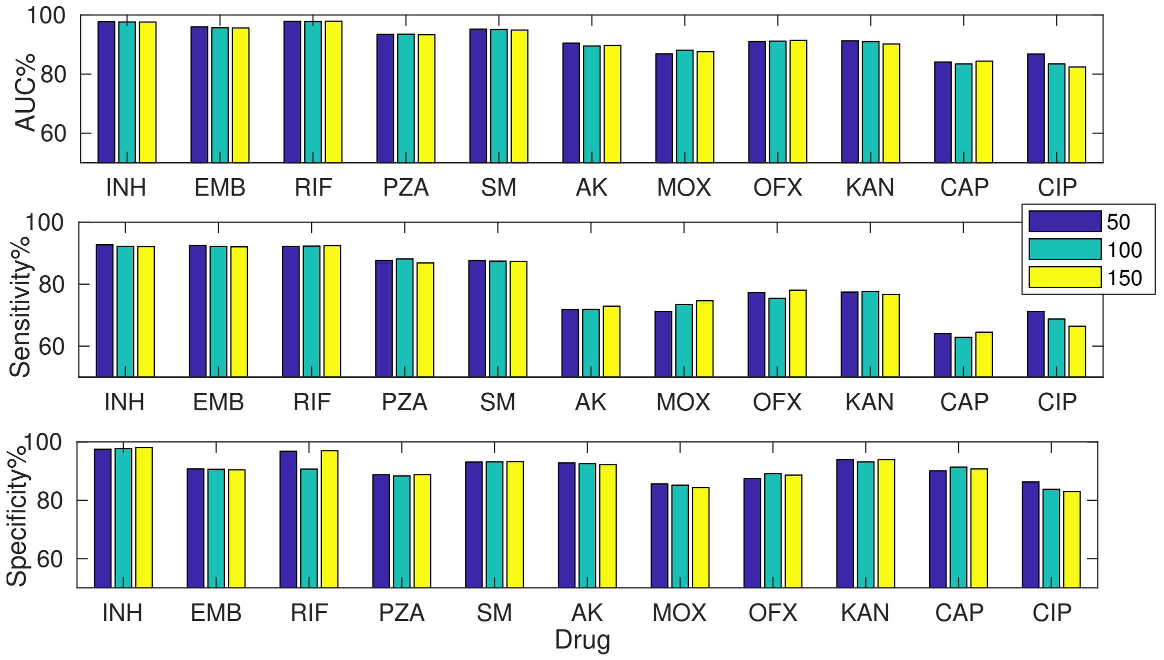

Fig. 7: Performance comparison in terms of AUC, sensitivity, and specificity considering 50, 100, and 150 components in (F1 + SNMF + GBT).

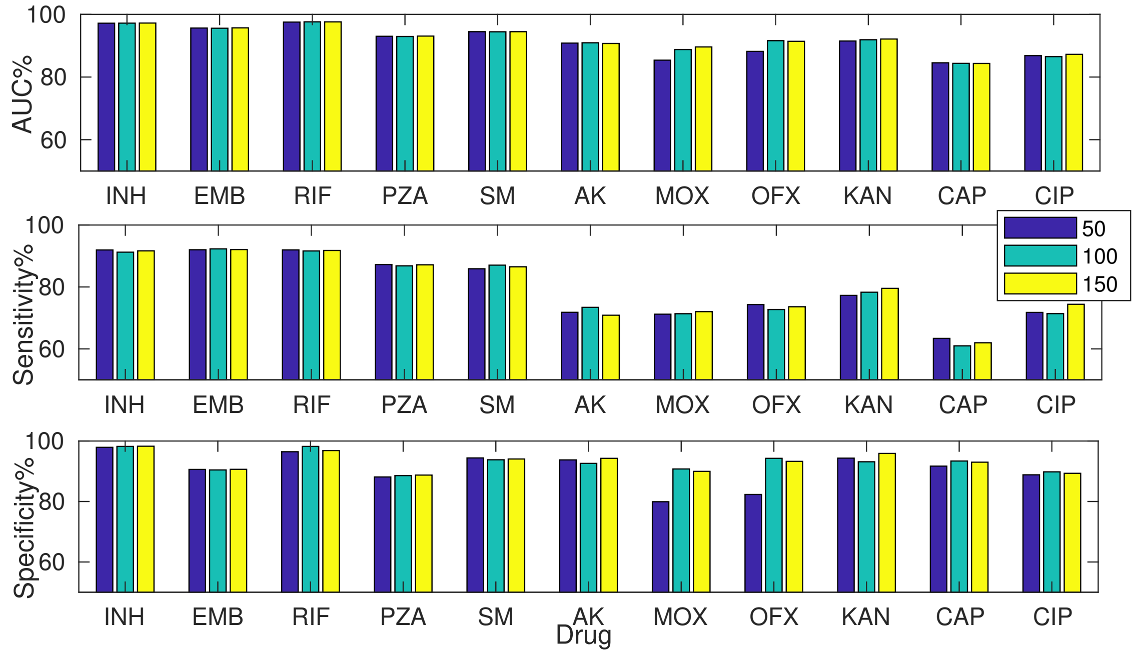

Fig. 8: Performance comparison in terms of AUC, sensitivity, and specificity considering 50, 100, and 150 components in (F1 + SNMF + GBT).

Supplementary K

The work presented by (Yang *et al.*, 2017) used the threshold that maximises the accuracy. There was no explanation on threshold setting in (Farhat *et al.*, 2016) but they set the RF parameters by maximising the sum of sensitivity and specificity.

Table 18. Comparing our best performance with the one reported in the work published by Yang et al. and Farhat et al.

	Our Performance			Yang et al			Farhat et al	
Drugs	Sensitivity	Specificity	AUC	Sensitivity	Specificity	AUC	Sensitivity	Specificity
INH	92.19 ± 0.94	98.38 ± 0.29	97.89 ± 0.38	97 ± 0.3	94± 0.4	99±0.0	96 ± 1	98 ± 2
EMB	92.12 ± 0.98	91.89 ± 0.84	96.25 ± 0.54	97 ± 1.0	96 ± 0.6	99 ± 0.1	84 ± 2	91 ± 2
RIF	92.27 ± 1.25	97.45 ± 0.63	98.08 ± 0.32	97± 0.4	97 ± 0.4	99± 0.1	93 ± 1	98 ± 1
PZA	88.12 ± 2.65	88.91 ± 1.66	93.89 ± 0.80	84 ± 1.2	90 ± 1.1	95 ± 0.2	72 ± 2	97 ± 1
SM	87.40 ± 1.98	94.15 ± 1.23	95.15 ± 0.56	87 ± 1.5	90 ± 1.0	91 ± 0.3	65 ± 2	97 ± 1
AK	77.23 ± 6.96	89.84 ± 3.05	91.37 ± 2.36	-	-	-	85 ± 3	98 ± 1
MOX	76.84 ± 9.29	87.19 ± 8.21	90.27 ± 2.96	95 ± 1.4	93 ± 1.0	95 ± 0.4	-	-
OFX	79.06 ± 6.94	90.88 ± 6.38	92.33 ± 1.49	96 ± 1.4	92 ± 1.3	95 ± 0.5	83 ± 5	90 ± 2
KAN	80.41 ± 6.48	93.48± 4.93	92.49 ± 2.93	-	-	-	66 ± 4	99 ± 0.5
CAP	64.44 ± 6.02	92.74 ± 2.52	85.46 ± 2.02	-	-	-	43 ± 3	96 ± 1
CIP	79.86 ± 9.98	85.37 ± 7.65	89.53 ± 4.06	96 ±0.9	98 ± 0.4	98 ± 0.3	56 ± 4	100 ± 0

Supplementary L

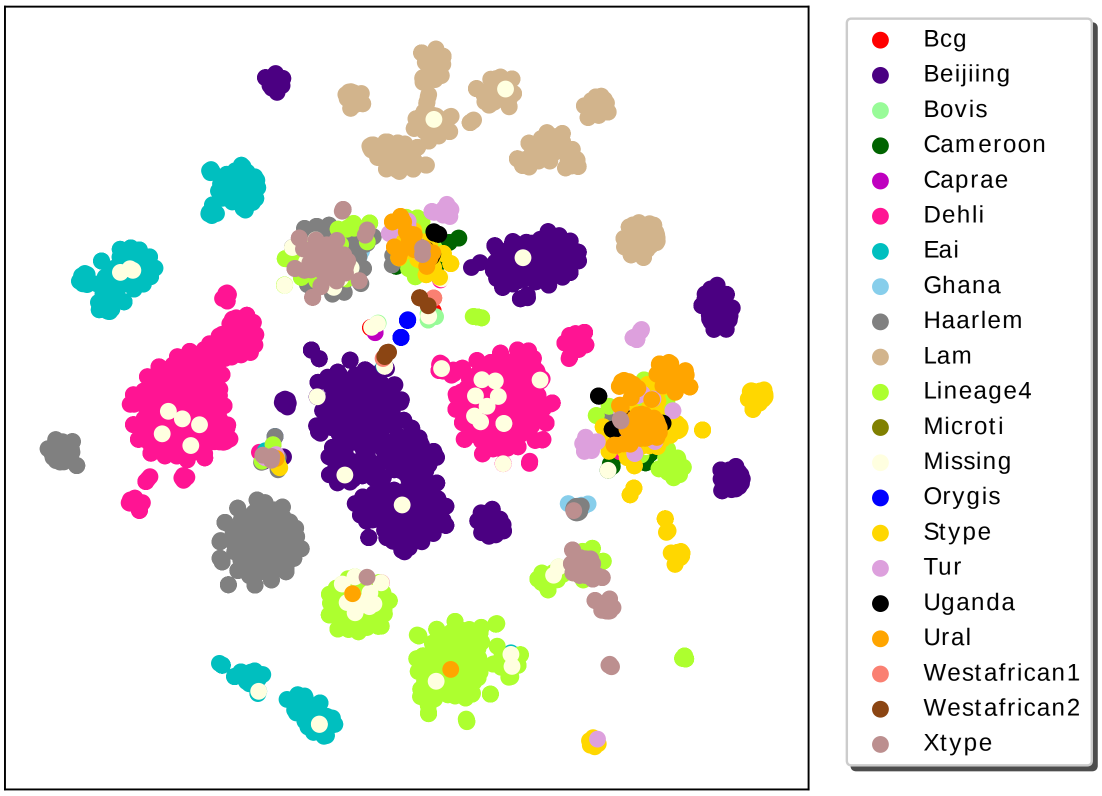

Fig. 9: t-distributed stochastic neighbour embedding (TSNE) visualisation of isolates coloured based on the associated lineage. It confirms isolates sharing the same lineage mainly form their own cluster. TSNE keeps the locality of the data while reducing the dimension and hence shows several lineages have patterns distinguishing them from others.

References

Farhat, M. R., Sultana, R., Iartchouk, O., Bozeman, S., Galagan, J., Sisk, P., Stolte, C., Nebenzahl-Guimaraes, H., Jacobson, K., Sloutsky, A., *et al.* (2016). Genetic determinants of drug resistance in mycobacterium tuberculosis and their diagnostic value. *American journal of respiratory and critical care medicine*, **194**(5), 621–630.

Yang, Y., Niehaus, K. E., Walker, T. M., Iqbal, Z., Walker, A. S., Wilson, D. J., Peto, T. E., Crook, D. W., Smith, E. G., Zhu, T., and Clifton, D. A. (2017). Machine learning for classifying tuberculosis drug-resistance from DNA sequencing data. *Bioinformatics*, **34**(10), 1666–1671.
